# Phenotypic, chemical and functional characterization of cyclic nucleotide phosphodiesterase 4 (PDE4) as a potential anthelmintic drug target

**DOI:** 10.1371/journal.pntd.0005680

**Published:** 2017-07-13

**Authors:** Thavy Long, Liliana Rojo-Arreola, Da Shi, Nelly El-Sakkary, Kurt Jarnagin, Fernando Rock, Maliwan Meewan, Alberto A. Rascón, Lin Lin, Katherine A. Cunningham, George A. Lemieux, Larissa Podust, Ruben Abagyan, Kaveh Ashrafi, James H. McKerrow, Conor R. Caffrey

**Affiliations:** 1 Center for Discovery and Innovation in Parasitic Diseases, University of California San Francisco, San Francisco, California, United States of America; 2 Department of Pathology, University of California San Francisco, San Francisco, California, United States of America; 3 Skaggs School of Pharmacy and Pharmaceutical Sciences, University of California San Diego, La Jolla, California, United States of America; 4 Anacor Pharmaceuticals, Inc., Palo Alto, California, United States of America; 5 Department of Physiology, University of California San Francisco, San Francisco, California, United States of America; McGill University, CANADA

## Abstract

**Background:**

Reliance on just one drug to treat the prevalent tropical disease, schistosomiasis, spurs the search for new drugs and drug targets. Inhibitors of human cyclic nucleotide phosphodiesterases (huPDEs), including PDE4, are under development as novel drugs to treat a range of chronic indications including asthma, chronic obstructive pulmonary disease and Alzheimer’s disease. One class of huPDE4 inhibitors that has yielded marketed drugs is the benzoxaboroles (Anacor Pharmaceuticals).

**Methodology/Principal findings:**

A phenotypic screen involving *Schistosoma mansoni* and 1,085 benzoxaboroles identified a subset of huPDE4 inhibitors that induced parasite hypermotility and degeneration. To uncover the putative schistosome PDE4 target, we characterized four PDE4 sequences (SmPDE4A-D) in the parasite’s genome and transcriptome, and cloned and recombinantly expressed the catalytic domain of SmPDE4A. Among a set of benzoxaboroles and catechol inhibitors that differentially inhibit huPDE4, a relationship between the inhibition of SmPDE4A, and parasite hypermotility and degeneration, was measured. To validate SmPDE4A as the benzoxaborole molecular target, we first generated *Caenorhabditis elegans* lines that express a cDNA for *smpde4a* on a *pde4(ce268)* mutant (hypermotile) background: the *smpde4a* transgene restored mutant worm motility to that of the wild type. We then showed that benzoxaborole inhibitors of SmPDE4A that induce hypermotility in the schistosome also elicit a hypermotile response in the *C*. *elegans* lines that express the *smpde4a* transgene, thereby confirming SmPDE4A as the relevant target.

**Conclusions/Significance:**

The orthogonal chemical, biological and genetic strategies employed identify SmPDE4A’s contribution to parasite motility and degeneration, and its potential as a drug target. Transgenic *C*. *elegans* is highlighted as a potential screening tool to optimize small molecule chemistries to flatworm molecular drug targets.

## Author summary

Just one drug, praziquantel (PZQ), is available to treat schistosomiasis, a flatworm disease that infects over 240 million people, mainly in Africa. With the expanding distribution of PZQ, and the associated threat of drug resistance, new drugs and drug targets are needed. We screened *Schistosoma mansoni* worms with over 1,000 benzoxaborole chemical molecules from Anacor Pharmaceuticals to identify a subset of human cyclic nucleotide phosphodiesterase 4 (huPDE4) inhibitors that cause parasite hypermotility and degeneration. We identified four PDE4 genes in the genome of the parasite and recombinantly expressed one of them (SmPDE4A) in bacteria. This enzyme was then used to uncover a relationship between the degree of enzyme inhibition, and the generation of parasite hypermotility and degeneration. To understand whether the SmPDE4A enzyme is the target of the benzoxaboroles in the parasite, we incorporated the coding DNA for SmPDE4A into the model nematode *Caenorhabditis elegans* that lacked its own functional PDE4 and, as a consequence, was hypermotile. These ‘transgenic’ worms displayed normal motility which could be increased by applying the most potent huPDE4 benzoxaborole inhibitors. In summary, the chemical, biological and genetic approaches taken identify SmPDE4A as a potential drug target for treating schistosomiasis. The potential value of *C*. *elegans* as a tool to test and optimize therapeutic chemistries for a flatworm disease is also highlighted.

## Introduction

Schistosomiasis, also known as bilharzia, is a ‘neglected’ tropical disease caused by the *Schistosoma* flatworm parasite that resides in the bloodstream. The disease is chronic and morbid, and affects more than 240 million people worldwide [1–3]. For over 35 years, treatment and control has relied on just one drug, praziquantel (PZQ) [4–6]. There are a number of ongoing international efforts that aim to increase the distribution of PZQ for mass drug administration [7, 8]. Consequently, there is concern regarding the possible emergence and establishment of drug resistance [5, 9–11]. Furthermore, PZQ has a number of pharmacological problems that encourage the search for alternate anti-schistosome therapies. The drug has diminished or no efficacy against developing schistosomes [12–15] and is rarely curative at the single 40 mg/kg dose employed [4, 16–18], in part due to its rapid metabolism [19, 20]. Also, the recommended dose used is high relative to other oral anthelmintics and medications in general, especially given its unpalatable taste [21] and that the primary target patient population is children.

Cyclic nucleotide phosphodiesterases (PDEs) [22–24] hydrolyse the second-messenger signalling molecules, cyclic adenosine monophosphate (cAMP) and cyclic guanosine monophosphate (cGMP) to produce 5’-AMP and 5’-GMP, respectively [24, 25]. Their activities contribute to the control of the intracellular concentrations of these ubiquitous cyclic nucleotides and influence signalling pathways in health and disease [23, 25–27]. In mammals, the PDE superfamily is divided into 11 families (PDE1–11) based of their sequence identity, biochemical and pharmacological properties, regulation and substrate specificity [23, 24, 28–30]. PDEs share a conserved C-terminal catalytic domain and have various N-terminal regulatory domains. Some PDEs hydrolyse cAMP or cGMP exclusively, whereas others hydrolyse both molecules [27–29, 31].

Among those PDEs that exclusively hydrolyse cAMP, the most extensively studied is the PDE4 multi-gene family with over 20 isoforms, each with a unique non-redundant role [32–35]. Due to their importance in angiogenesis, neuronal function, and immune and inflammatory stress responses, PDE4s have attracted considerable attention over the past decade as drug targets and selective inhibitors have shown promise in *in vitro* and *in vivo* models of asthma, depression, and Parkinson’s and Alzheimer’s diseases [23, 35–40]. Currently, first generation PDE4-selective inhibitors, such as rolipram, and second generation inhibitors, such as roflumilast and cilomilast, are used to treat chronic obstructive pulmonary disease [41–43]. For cognitive decline and Alzheimer’s disease, PDE4 inhibitors are under investigation in animal models and in the clinic [44–48].

In relation to parasitic (protozoal) diseases, PDEs, including PDE4 and their inhibitors, have been investigated for their therapeutic potential [49–51]. For example, *Trypanosoma brucei* expresses five PDEs [52] of which TbrPDEB1 and TbrPDEB2 are confirmed druggable targets [53–56]. The chemical validation of TbrPDEB1 and TbrPDEB2 as targets has been performed using phenylpyridazinones [57, 58] and a series of catechol pyrazolinones [59]. In *Leishmania*, PDEs are also considered valuable therapeutic targets [60, 61] given their contributions to the regulation and compartmentalization of cAMP signaling, processes that are essential for parasite transformation, differentiation and proliferation. In *Trypanosoma cruzi*, an ortholog of huPDE4, TcPDE4 (TcPDEB1) displayed an inhibition profile characteristic of the PDE4 subfamily, including a specificity for cAMP over cGMP [62]. Lastly, PDE inhibitors block the *in vitro* proliferation of *Plasmodium falciparum* and *Toxoplasma gondii* [63, 64].

For the schistosome parasite, the function(s) and potential of PDE4 as a drug target are unknown. G-protein-coupled receptors, adenylyl cyclases (AC) and protein kinase A (PKA) have been characterized in *S*. *mansoni* suggesting that the parasite possesses a functional cAMP signaling pathway [65, 66]. For the snail-infective miracidial stage of *S*. *mansoni*, both cAMP and a cAMP-dependent protein kinase have been chemically shown to control ciliary motion [67] and treatment of miracidia with AC modulators [68, 69] inhibited transformation of miracidia to mother sporocysts. Furthermore, exposure of miracidia to the general PDE inhibitor, IBMX, delayed transformation [69].

From a phenotypic screen involving *Schistosoma mansoni* and 1,085 benzoxaboroles (Anacor Pharmaceuticals), we identified a subset of human (hu)PDE4 inhibitors that induced hypermotility and degeneration. Benzoxaboroles, which incorporate a boron atom into the 5-membered ring of a six-five bicyclic molecule, are a new class of versatile drugs and drug candidates that interact with a variety of enzymes. Thus, the drug, tavaborole, which inhibits aminoacyl-tRNA synthetase [70], has been approved for treatment of onychomycosis [71]; also, tavaborole derivatives have been developed as anti-bacterial candidates [72]. In addition, crisaborole, which inhibits human (hu)PDE4 [73], has completed clinical trials for treatment of atopic dermatitis [74, 75]. Finally, SCYX-7158 has completed Phase I clinical testing as an oral, single dose cure of Human African Trypanosomiasis [76]. A phase II/III trial is now underway with recruitment of patients in the Democratic Republic of Congo [77].

Based on data from the phenotypic screen, we followed up by recombinantly expressing the orthologous *S. mansoni* PDE4 enzyme, SmPDE4A, and uncovered an association between enzyme inhibition and anti-parasite activity. Given the challenges of genetically manipulating the schistosome [78], we employed *Caenorhabditis elegans* to understand whether the parasite gene could functionally replace the nematode’s endogenous *pde4* gene, and whether that transgene is the target of the anti-schistosomal benzoxaborole inhibitors.

## Results

### Screening of *S*. *mansoni* somules with a benzoxaborole library identifies a subset of huPDE4 inhibitors that induce hypermotility and degeneration

**Fig 1:**
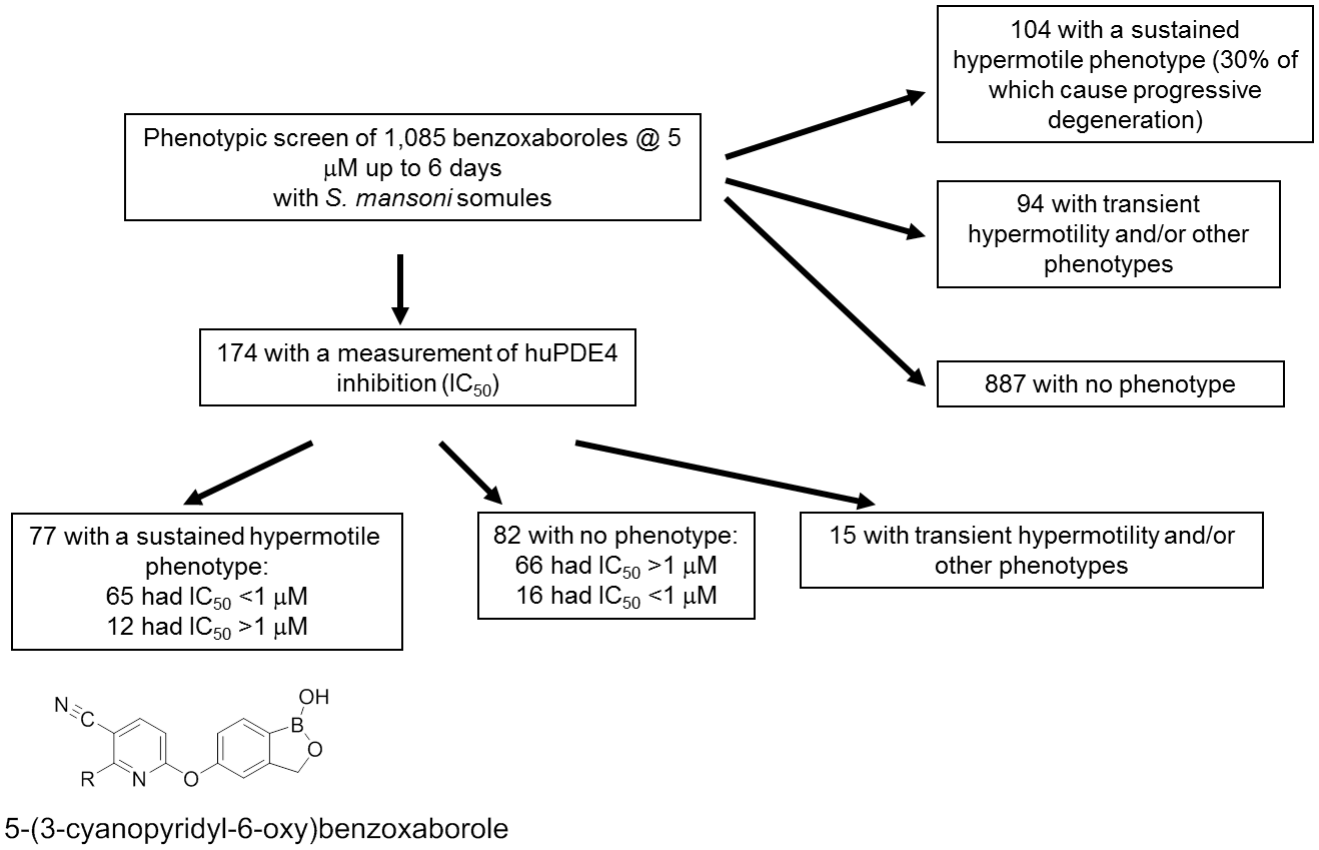
Phenotypic screening of a benzoxaborole collection with *S*. *mansoni* somules suggests a PDE4 as a molecular target of interest. The screen involving 1,085 benzoxaboroles was performed at 5 μM for 6 days with observations taken every day using a constrained nomenclature, as noted in the text. Three main phenotype response groups could be adjudicated by microscopical observation: (i) 104 compounds eliciting an early and sustained hypermotile phenotype, of which, 30% was also associated with a parasite degeneration; (ii) 94 compounds that yielded a range of phenotypic responses (*e*.*g*., rounding, darkening), including hypermotility, which were either transient (noted at 24 h only) or appeared later (on or after day 3 of the incubation), and (iii) 887 compounds that produced no phenotype. Of the 1,085 benzoxaboroles screened, 174 also had IC_50_ data for inhibition of huPDE4B2 that were distributed as 77, 82 and 15 compounds across the sustained hypermotile, no phenotype and transient hypermotile groups, respectively. Sixty-five of 77 compounds in the sustained hypermotile group inhibited huPDE4B2 with IC_50_ values of < 1 μM. In contrast, for the no phenotype group, only 16 of 82 compounds had IC_50_ values of < 1 μM. The association between the sustained hypermotile phenotype and sub-micromolar inhibition of huPDE4 was highly significant with a Fishers exact *p*-value of <0.0001. The 5-(3-cyanopyridyl-6-oxy) benzoxaborole scaffold, which is known to preferentially inhibit huPDE4 over other PDEs [73], was well represented in Group 1.

A 5 μM single concentration screen of *S*. *mansoni* somules (schistosomula) over 6 days with a collection of 1,085 benzoxaboroles identified three phenotype response groups as judged by microscopical observation (Fig 1): (i) 104 compounds eliciting an early and sustained hypermotile phenotype, of which, 30% was associated with a progressive degeneration of the parasite; (ii) 94 compounds that yielded a range of phenotypic responses (*e*.*g*., rounding, darkening), including hypermotility, which was either transient (noted at 24 h only) or appeared later in the incubation period (on or after day 3), and (iii) 887 compounds that yielded no phenotype.

Of the 1,085 benzoxaboroles phenotypically screened, 174 also had associated IC_50_ data for inhibition of huPDE4B2 (Fig 1) that were distributed as 77, 82 and 15 compounds across the sustained hypermotile, no phenotype and transient hypermotile groups, respectively. Of the 77 compounds in the sustained hypermotile group, 65 had IC_50_ values for inhibition of huPDE4B2 of < 1 μM. In contrast, for the 82 compounds with no phenotype, only 16 had IC_50_ values of < 1 μM. The association between the sustained hypermotile phenotype and sub-micromolar inhibition of huPDE4 was highly significant with a Fishers exact *p*-value of <0.0001. Also, the 5-(3-cyanopyridyl-6-oxy) benzoxaborole scaffold, which is known to preferentially inhibit huPDE4 over other PDEs [73], was enriched in compounds that caused the sustained hypermotile phenotype (Fig 1). In sum, therefore, the phenotypic and associated biochemical data focused our attention on identifying a schistosome PDE4 and understanding whether engagement of that enzyme was associated with the hypermotility (and degeneracy) recorded.

### *S*. *mansoni* possesses four PDE4-like genes

**Fig 2:**
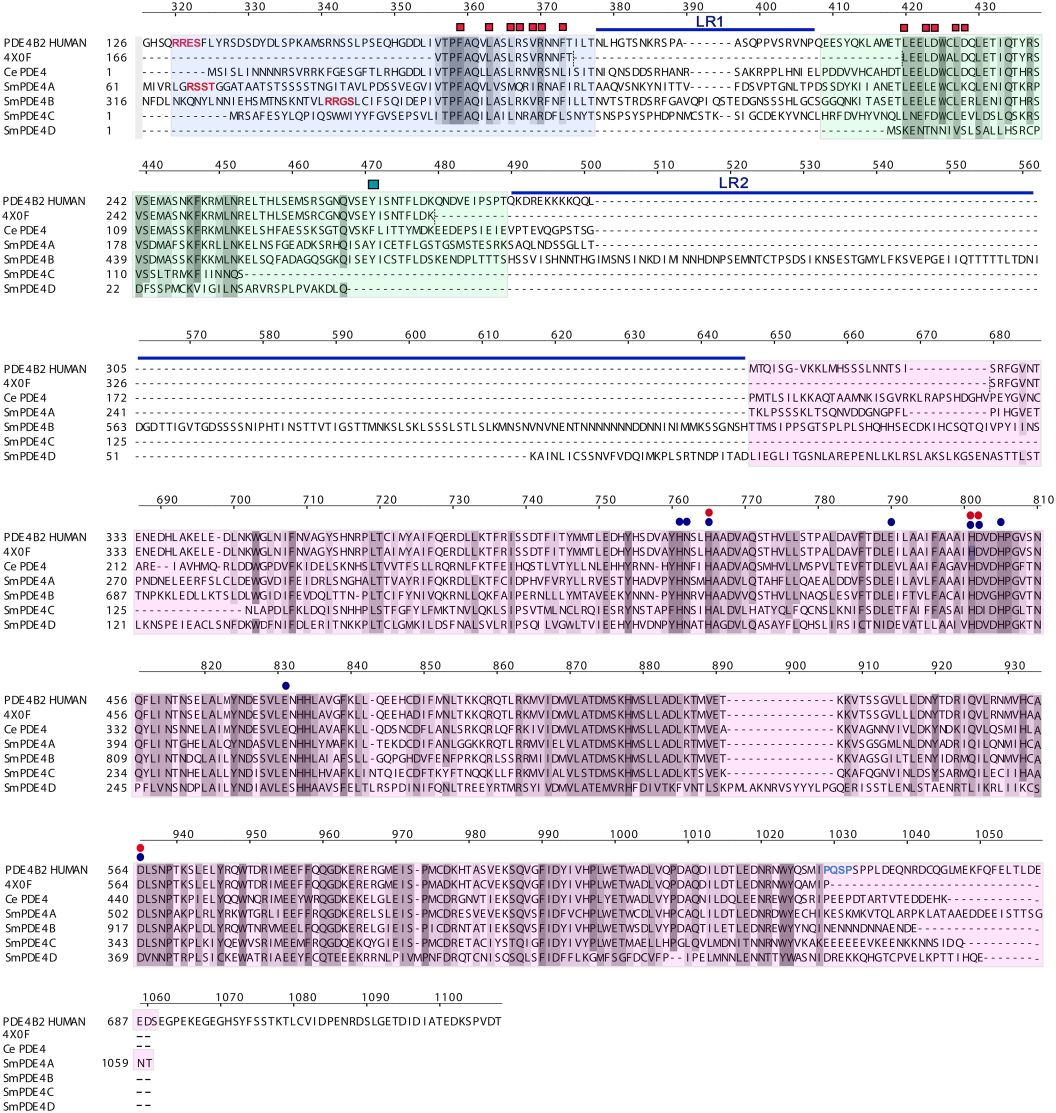
Protein sequence alignment of human, *Caenorhabditis elegans* and *Schistosoma mansoni* PDE4 sequences. Shown are the sequences for huPDE4B2 (NP_001032416.1), the crystal structure of a crosslink-stabilized huPDE4B1 (4X0F) [79], *C*. *elegans* (NP_495601.1) and *S*. *mansoni* Smp_134140 (PDE4A), Smp_141980 (PDE4B), Smp_129270 (PDE4C) and Smp_044060 (PDE4D). Upstream Conserved Regions (UCR) 1 and 2, and the catalytic domain are shaded in blue, green and pink, respectively. Demarcations of these domains are according to [80]. Linker regions (LR) 1 and 2 are indicated by blue horizontal bars. If present, the PKA and ERK phosphorylation sites, in the UCR1 and the catalytic domain, respectively, are indicated by the red and blue typeface, respectively. The conserved PDE signature motif HNX_2_HNX_N_E/D/QX_10_HDX_2_HX_25_E is indicated with blue circles and those residues that coordinate directly with the catalytic zinc in the substrate binding pocket are also indicated by red circles [33, 93]. The residues in UCR1 and UCR2 that contribute to the dimerization interface in huPDE4 [79] are indicated by red squares and the tyrosine residue (Y471 (Y274 in [79]) in UCR2 that contributes to the high affinity binding of rolipram in the PDE4 active site is marked by the teal square. The sequence alignment is adjusted N-terminally given the very long N-termini in some cases, *e*.*g*., for SmPDE4B. The alignment was performed semi-automatically using the ICM pro (Molsoft LLC).

To identify orthologs of huPDE4 in *S*. *mansoni*, we employed the huPDE4B2 (NP_001032416.1) in a BlastP analysis constrained to taxid ID: 6183 (*Schistosoma mansoni*). We retrieved four PDE4-like protein sequences, namely Smp_134140 (CCD81292.1; 626 amino acids in length), Smp_141980 (CCD80549.1; 1,022 amino acids), Smp_129270 (454 amino acids) and Smp_044060 (CCD77807.1; 482 amino acids). We term these sequences SmPDE4A through SmPDE4D, respectively. A protein sequence alignment of the *S*. *mansoni* sequences against huPDE4B2 (NP_001032416.1), the recently published sequence used to generate a crystal structure of huPDE4B1 (PDB ID: 4X0F) [79] and the *C*. *elegans* ortholog (NP_495601.1) confirmed their homology with the PDE4 family (Fig 2).

Among huPDEs, PDE4 has unique sequence features upstream of the catalytic domain, namely Upstream Conserved Regions (UCR)1 and 2, each of which is succeeded by a Linker Region (LR1 and 2; Fig 2). The presence or part absence of these UCRs characterizes the three principal huPDE4 variants. Thus, PDE4 ‘long isoforms’ contain both UCRs; ‘short isoforms’ lack the UCR1 and ‘super short isoforms’ contain an N-terminally-truncated UCR2 [34, 79–81]. As a general rule, long isoforms act as dimers whereas short forms are monomers [82]. Dimerization is facilitated via both UCRs [28, 83] and in an engineered construct of huPDE4B2, the dimerization domain comprises the C-terminus of UCR1 and the N-terminus of UCR2 which form an antiparallel helix pair [79].

Based on the sequence alignment (Fig 2), huPDE4B2, SmPDE4A-C and the *C*. *elegans* ortholog share obvious homology with the human enzyme in the last one-third of UCR1: SmPDE4D has no UCR1. Downstream of LR1, UCR2 is better conserved across all of the sequences except for SmPDE4C which is missing approximately the C-terminal half of the region as well as LR2 and approximately 60 amino acids at the N-terminus of the catalytic domain. The catalytic domain itself is well-conserved across all of the sequences, except, as just indicated for SmPDE4C; also, SmPDE4D has a 16 amino acid insert at position 892 (Fig 2). SmPDE4B stands out in possessing the longest N-terminal sequence (~315 amino acids) upstream of UCR1 and a large insert (~156 amino acids) between UCR2 and the catalytic domain.

The catalytic domain of the PDE4 sequences examined possesses the conserved PDE signature motif HNX_2_HNX_N_E/D/QX_10_HDX_2_HX_25_E [84] and the four key residues that coordinate with the catalytic zinc cation (H765, H801, D802 and D935) [28, 33, 85] (Fig 2).

For huPDE4, UCR1 regulates phosphohydrolase activity via a R-R-E-S variant of the R-X-X-S/T phosphorylation consensus motif for protein kinase A (PKA; Fig 2) which increases PDE4 activity and results in enhanced cAMP degradation [86–88]. This site is present in SmPDE4A and B but absent in the other helminth orthologs (Fig 2). Absent in all of the helminth sequences is a P-X-S/T-P consensus motif for ERK phosphorylation near the C-terminus of the catalytic domain [89–91]. The functional consequences of ERK phosphorylation are dependent on the presence of UCR1 and UCR2 such that long isoforms are catalytically inhibited whereas short isoforms have increased activity, and super-short isoforms are again weakly inhibited [33, 91]. The absence of PKA and ERK phosphorylation sites in some or all the helminth orthologs suggest differences in how the respective proteins are regulated relative to mammalian PDE4s. SmPDE4A was chosen for recombinant expression and subsequent enzyme activity/inhibition studies as it was the least divergent in its protein sequence and domain organization from huPDE4, which has been successfully expressed by Anacor for its own drug development programs [92].

### SmPDE4 genes are expressed in various developmental stages of *S*. *mansoni* with some orthologs being present in the genomes of *Schistosoma haematobium* and *Schistosoma japonicum*

Querying the GeneDB database reveals that all four SmPDE4 enzymes are expressed in a number of different developmental stages of *S*. *mansoni* relevant to infection in humans (cercariae, somules, and adult male and/or female worms; S1 Table). NCBI BLAST analysis of the genomes of *S*. *haematobium* [94] and *S*. *japonicum* [95], indicates that orthologs of SmPDE4A, B and D (not C) are present in adult *S*. *haematobium*, and that orthologs of SmPDE4A and B (not C or D) are found in the adult male and somules of *S*. *japonicum*, respectively (S1 Table and S1–S3 Figs for alignments).

Each of the SmPDE4 genes shares greatest homology with its respective ortholog in *S*. *haematobium* over the full sequence (83–90%; S2 Table) or the catalytic domain (87–98%; S3 Table). For SmPDE4A and B, the corresponding ortholog identities *in S*. *japonicum* are generally lower, 63 and 34% for the full sequence, and 89 and 35% for the catalytic domain. For either the full length sequence or that of the catalytic domain, the percentage identities with huPDE4B2 are approximately 60% for SmPDE4A and SmPDE4B, 50% for SmPDE4C and 34% for SmPDE4D. Similar data were obtained for the same comparisons between the SmPDE4 and the *C*. *elegans* sequences.

### Recombinant expression and catalytic activity of SmPDE4A

**Fig 3:**
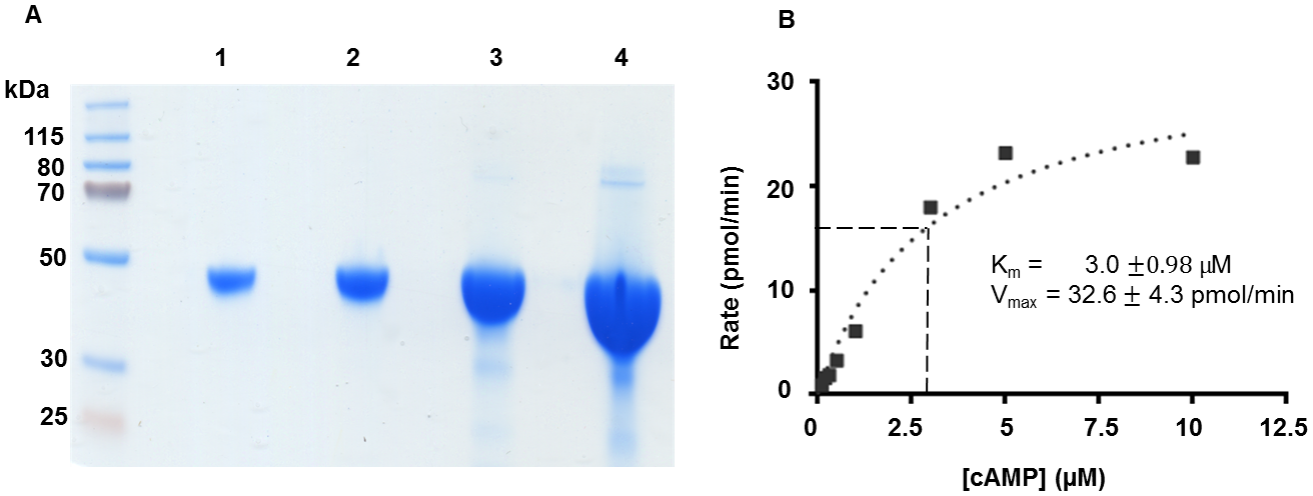
Purification and activity analysis of the recombinant catalytic domain of SmPDE4A. (**A**) The three-step purification scheme described in the text resulted in purified His_6_-tagged SmPDE4A with the expected molecular mass of 44.2 kDa. Each lane (1–4) contains an increasing amount of protein (3, 6, 21 and 59 μg) demonstrating the absence of major contaminants. Molecular mass markers (in kDa) are indicated on the left. (**B**) Determination of K_m_ and V_max_. Enzyme reaction rates were measured over increasing concentrations of the cAMP substrate up to 10 μM. All reactions ran for six minutes and contained 23.5 units/ml SmPDE4A. K_m_ and V_max_ values were determined by nonlinear regression analysis of the data (Prism GraphPad version. 6.03) using a Michaelis-Menten enzyme kinetics model. All data points were determined in triplicate.

The three-step purification scheme involving metal-ion affinity chromatography, hydrophobic interaction chromatography and Mono Q ion-exchange chromatography yielded a single purified protein with the expected molecular mass of 44.2 kDa (Fig 3A). Starting with approximately 55 g bacterial paste from a 6 L culture, 8.2 mg of purified His_6_-tagged SmPDE4A was obtained. Phosphohydrolase activity was measured using [^3^H]-cAMP as described [96]. The recombinant enzyme displayed Michaelis-Menten kinetics with a Michaelis constant (K_m_) of 3.0 μM and a maximum velocity (V_max_) of 32.6 pmol/min (Fig 3B). The K_m_ value is similar to values of 0.98, 2.25 and 7.81 μM reported previously for huPDE4A, B and D, respectively [96].

### SmPDE4 inhibition potency is associated with anti-schistosomal activity

**Fig 4:**
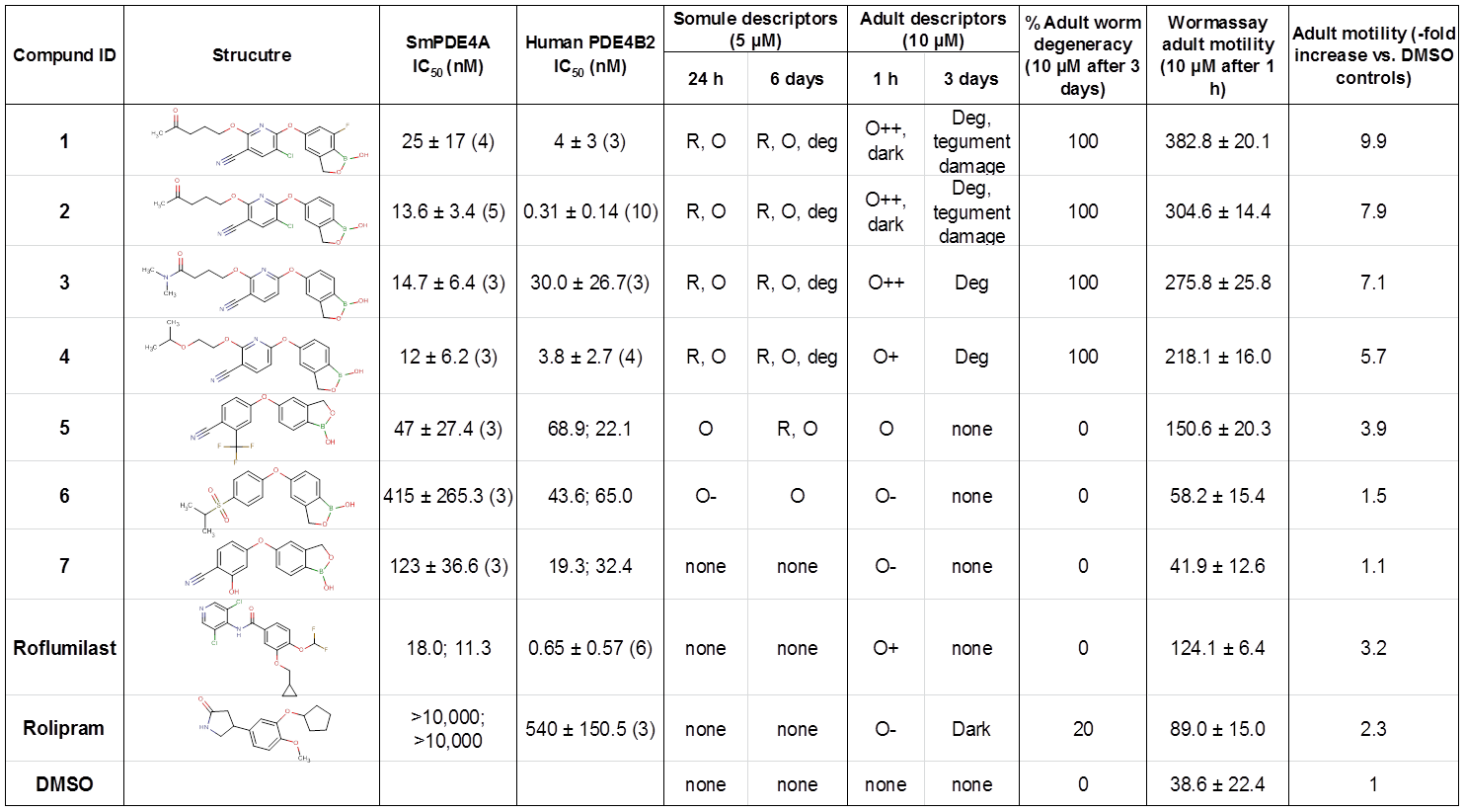
Association between inhibition of PDE4 and activity against the parasite for benzoxaborole and catechol inhibitors. Assays to determine IC_50_ values were performed in duplicate with the total number of assays performed indicated in parentheses: at a minimum, data from two assays are shown. Descriptions of phenotypes observed (descriptors): R = rounded; O = overactive (and perceived degrees thereof using plus and minus symbols); deg = degeneration; tegument damage = damage to the surface of the worms, dark = worms are darkened; none = no effects observed. Wormassay [102] is a digital camera based assay that detects adult worm-induced changes in the occupation and vacancy of pixels between frames (outputted as an average ± S.D.).

The phenotypic screen of 1,085 benzoxaboroles with *S*. *mansoni* somules had identified a cluster of compounds that induced sustained hypermotility and, in 30% of those cases, degeneracy. For 77 of these compounds for which huPDE4B2 inhibition data were also available to Anacor, the majority (65) had sub-micromolar IC_50_ values. The underlying implication was, therefore, that a schistosome PDE4 may be associated with the phenotypes observed. Accordingly, we selected benzoxaboroles (compounds **1**–**7**) with various peripheral substitutions to understand whether an association between enzyme inhibition and anti-parasite activity could be measured (Fig 4). Compounds were selected on the bases of (i) availability, (ii) existence of IC_50_ values for huPDE4 and (iii) absence of IP-constraints to reveal structures. The analysis also included two catechol drugs that inhibit huPDE4, namely, rolipram and roflumilast [41].

**Fig 5:**
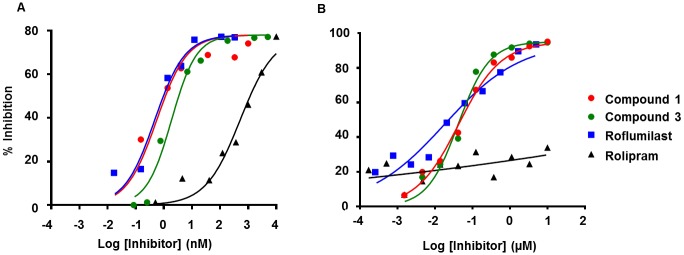
Inhibition of huPDE4B2 and SmPDE4A by exemplar benzoxaborole and catechol inhibitors. Assays with each inhibitor were performed using the catalytic domains of (**A**) huPDE4B2 (NP_001032416.1) and (**B**) SmPDE4A (Smp_134140). Briefly, each reaction contained 23.5 units/ml SmPDE4A or 30 pg/ml huPDE4B2. Assays were performed in triplicate, and IC_50_ values were determined by non-linear regression analysis using the four-parameter logistic equation (Prism GraphPad version. 6.03). Compound structures are shown in Fig 4.

IC_50_ values for the selected benzoxaboroles and catechols were determined for the recombinant SmPDE4A and compared to Anacor’s in-house data for huPDE4B2 (Fig 4); see Fig 5 for representative IC_50_ curves). For SmPDE4A, the most potent benzoxaborole inhibitors (**1**–**5**) containing p-cyano and 2-oxy substitutions yielded IC_50_ values of < 50nM. Compounds **6** and **7** with 3-sulfone and 2-hydroxy substitutions were less effective (415 and 123 nM, respectively). The catechol, roflumilast, was an effective inhibitor (IC_50_ = 18 and 11 nM), whereas rolipram was ineffective (IC_50_ >10 μM). For huPDE4B2, a similar trend was noted: compounds **1**–**4** yielded IC_50_ values of ≤ 30nM, whereas the values for **5**–**7** ranged between 19 and 69 nM. The catechol, roflumilast, was a potent inhibitor (IC_50_ = 0.65 nM) and rolipram much less so (IC_50_ = 540 nM) but still at least 19-fold more effective than against SmPDE4A (Fig 4). Inhibition values obtained for these two catechols against huPDE4 are consistent with those previously reported [79, 97, 98].

For the nine compounds, bioactivity as a function of time against both somules (at 5 μM) and adult *S*. *mansoni* (at 10 μM) was recorded observationally using our constrained nomenclature [99–101]: for adults, we also used Wormassay [102] to measure motility (Fig 4). Those most potent inhibitors of SmPDE4A were also the most bioactive against the parasite irrespective of developmental stage. Thus, by the first time point of 24 h for somules and 1 h for adults, compounds **1**–**4** induced intense hypermotility, which by day 6 for somules and day 3 for adults had progressed to include severe degenerative changes (Fig 4). For both somules and adults, degeneration appeared to occur throughout the worm body (not localized to a particular region or feature) and was irreversible upon removal of the inhibitors after the respective incubation periods employed (see S4 Fig for examples of adult parasites after incubation with compound **2**). In support of the intense hypermotility observed for adults after 1 h in the presence of compounds **1**–**4**, motility as measured by Wormassay was 6-10-fold greater than that of the DMSO control. For the less potent inhibitors (**5**–**7**) of SmPDE4A, bioactivity, if observed at all, was restricted to mild and/or transient increase in motility (for adults a maximum 3.9-fold over DMSO controls by Wormassay) without any associated degeneration. Finally, the catechols were inactive against somules and only induced transient hypermotility in adults (maximum 3.2-fold by Wormassay for roflumilast) without major degenerative changes. Overall, therefore, there appears to be a reasonable association between the potency of inhibition of SmPDE4A and the degree of hypermotility of the parasite, which at its most extreme, is associated with degenerative changes.

### Functional phenotypic rescue of pde4-deficient mutant of *C*. *elegans* by expression of SmPDE4A cDNA

**Fig 6:**
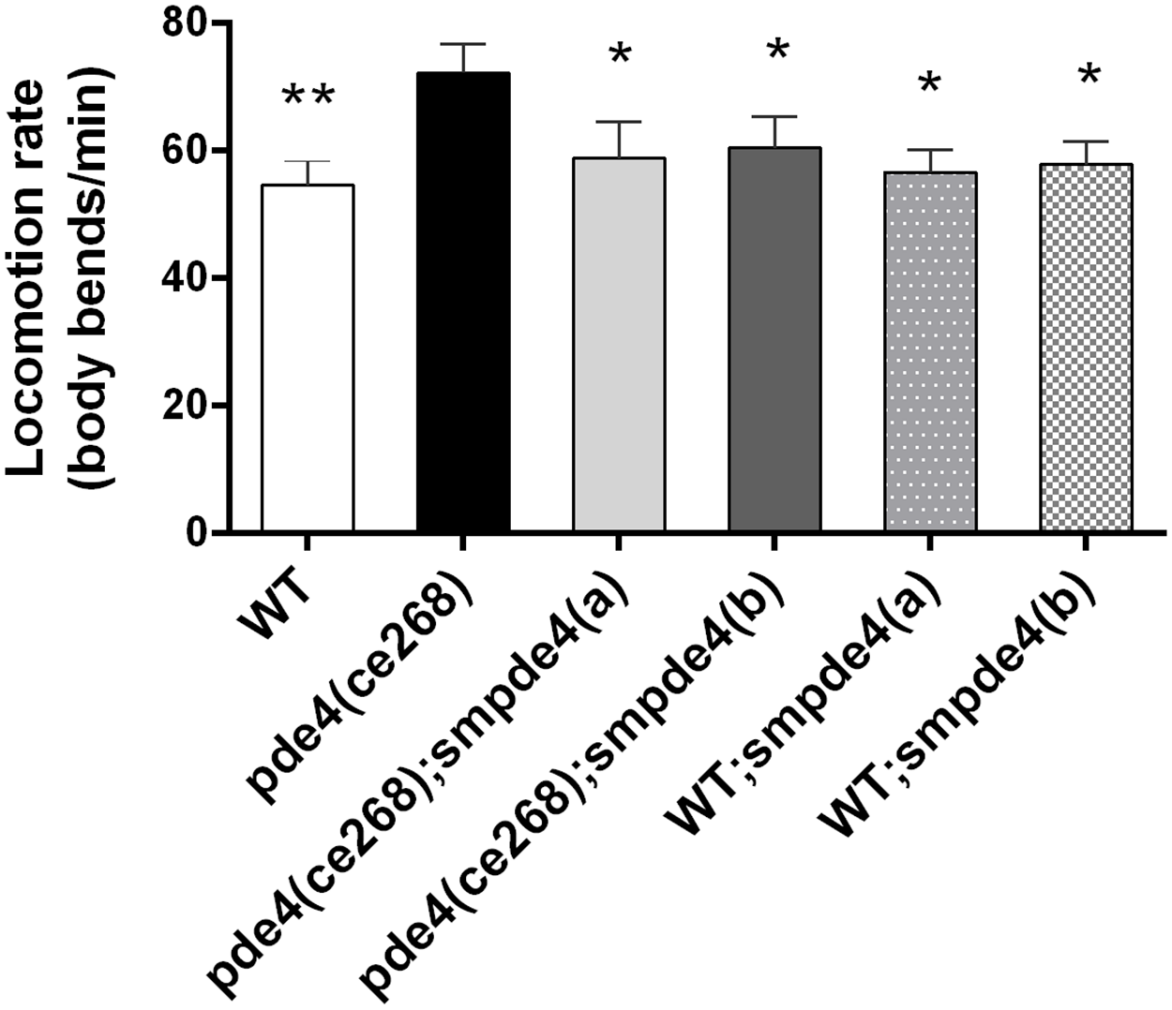
A *smpde4A* transgene restores wild type motility rates to *pde4*-deficient *C*. *elegans*. Relative to wild type (WT) *C*. *elegans*, a loss of function allele of *pde4*, namely *ce268* [103], causes hypermotility. This hypermotility is reverted back to WT rates upon transgenic expression of a full-length cDNA for *smpde4a*. Results for two independently generated lines, *smpde4a(a)* and *smpde4a(b)* are shown. The same transgenes do not alter the motility of otherwise WT animals. Error bars indicate the standard deviations around the mean motility in a representative experiment with at least 10 worms for each strain. The asterisks indicate significance by Student’s *t*-test (*p<0.005; **p<0.0005) relative to the hypermotility recorded for the *pde4(ce268)* mutant.

In the model nematode, *C*. *elegans*, a single PDE4 gene is responsible for maintaining normal motility such that disruption of that gene (*ce268* mutation) results in hypermotility [103]. Hypermotility of this *C*. *elegans* mutant is thought to be due to excessive cAMP accumulation and consequent hyper-activation of signaling pathways that promote motility [103]. To investigate whether the SmPDE4A can functionally substitute for the *C*. *elegans pde4*, we generated transgenic *C*. *elegans* that express full-length SmPDE4A under a pan-neuronally expressed promoter. In two independently generated transgenic lines of *C*. *elegans* that express *smpde4a* in the *C*. *elegans pde4(ce268)* mutant background, *smpde4a(a)* and *smpde4a(b)*, we found that the *S*. *mansoni* transgene restored normal motility rates (Fig 6). This was not simply due to non-specific motility-reducing effects of these transgenes as the same two transgenes did not affect the motility rates of otherwise wild type (WT; N2 Bristol strain) animals (Fig 6). Thus, the function of the endogenous *C*. *elegans pde4* gene can be complemented by the *smpde4a* transgene.

### PDE4 inhibitors act via the endogenous *pde4* or the *smpde4a* transgene to induce hypermotility in *C*. *elegans*

**Fig 7:**
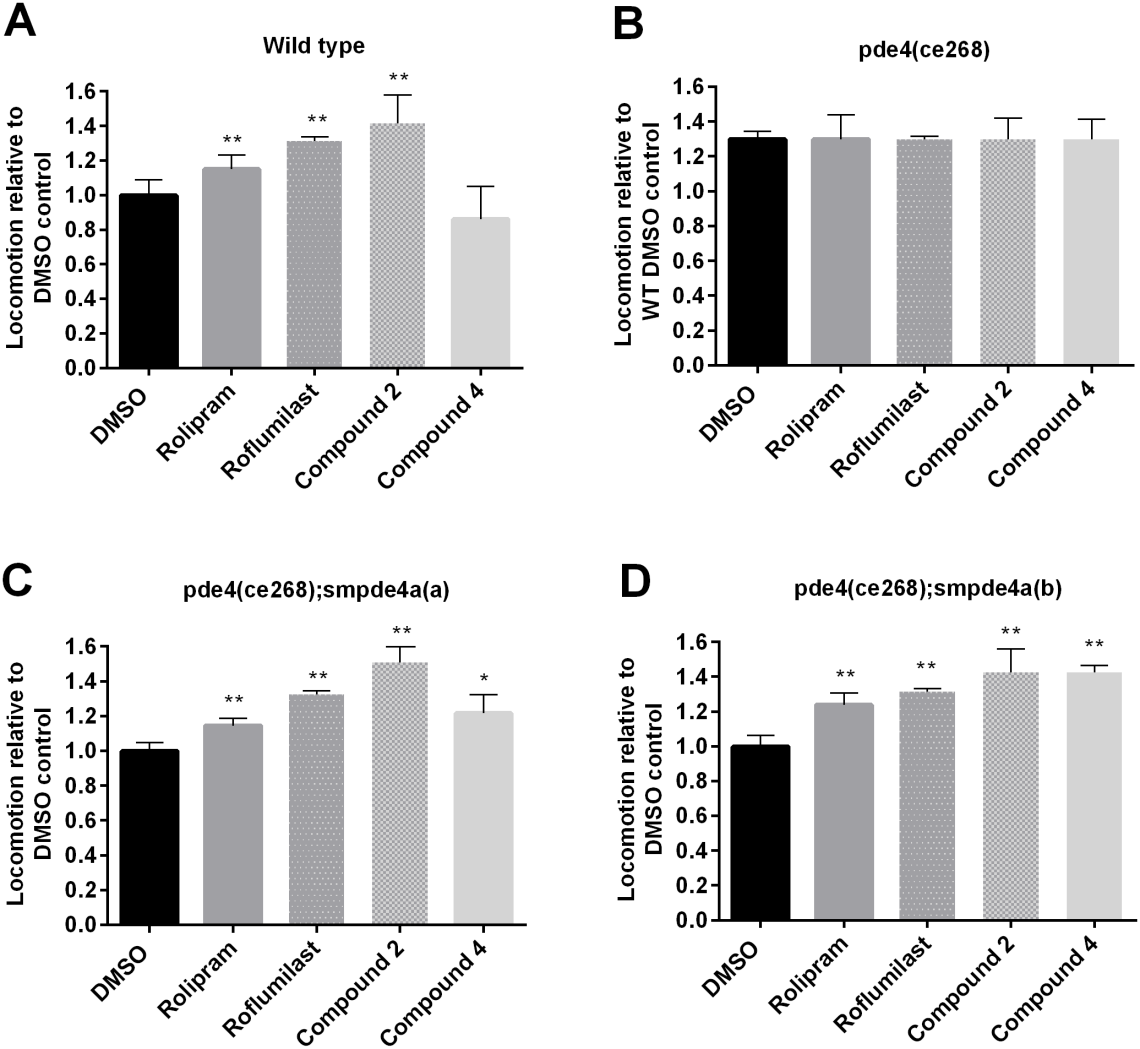
PDE4 inhibitors induce hypermotility in transgenic *C*. *elegans* expressing *smpde4a*. *C*. *elegans* was exposed to 100 μM rolipram, roflumilast or the benzoxaborole compounds **2** and **4**, and then worm motility measured. Effects on (**A**) WT, (**B**) hypermotile mutant *pde4(ce268)*, (**C**) transgenic *pde4(ce268);smpde4a(a)* and (**D**) transgenic *pde4(ce268);smpde4a(b)* are shown. Means and standard deviations for motility are normalized over two experiments to DMSO controls; each experiment involved measuring at least 10 worms per treatment. The asterisks in each panel indicate significance by Student’s *t*-test (*p<0.005; **p<0.0005) relative to the respective DMSO controls. For Panel B, motility was normalized to that recorded for the WT control.

We first asked whether exemplar PDE4 inhibitors (compounds **2** and **4**) could induce hyper-motility in WT *C*. *elegans*. Exposure of WT *C*. *elegans* to rolipram, roflumilast and compound **2** increased motility relative to the DMSO control whereas compound **4** did not (Fig 7A). Consistent with the notion that these compounds cause hypermotility through inhibition of PDE4, the already elevated motility of *pde4* mutant *C*. *elegans* was not further modulated by exposure to any of the PDE4 inhibitors (Fig 7B).

Exposure of the two mutant *C*. *elegans* lines carrying the *smpde4a* transgene to each of the PDE4 inhibitors induced hypermotility (Fig 7C and 7D) indicating that the compounds act via the schistosome transgene. The differential effects of compound **4** on WT and transgenic *C*. *elegans* may indicate differences between the susceptibilities of the *C*. *elegans* and *S*. *mansoni* PDE4 target enzymes to inhibition by this compound. Interestingly, rolipram, which was a weak inhibitor of recombinant SmPDE4A (Fig 4), increased motility significantly in both mutant *C*. *elegans* lines, albeit less so than the hypermotility induced by compounds **2** and **4** (Fig 7C and 7D).

### Differences in the inhibition of huPDE4B and SmPDE4A by catechols is associated with particular residues surrounding the binding site

**Fig 8:**
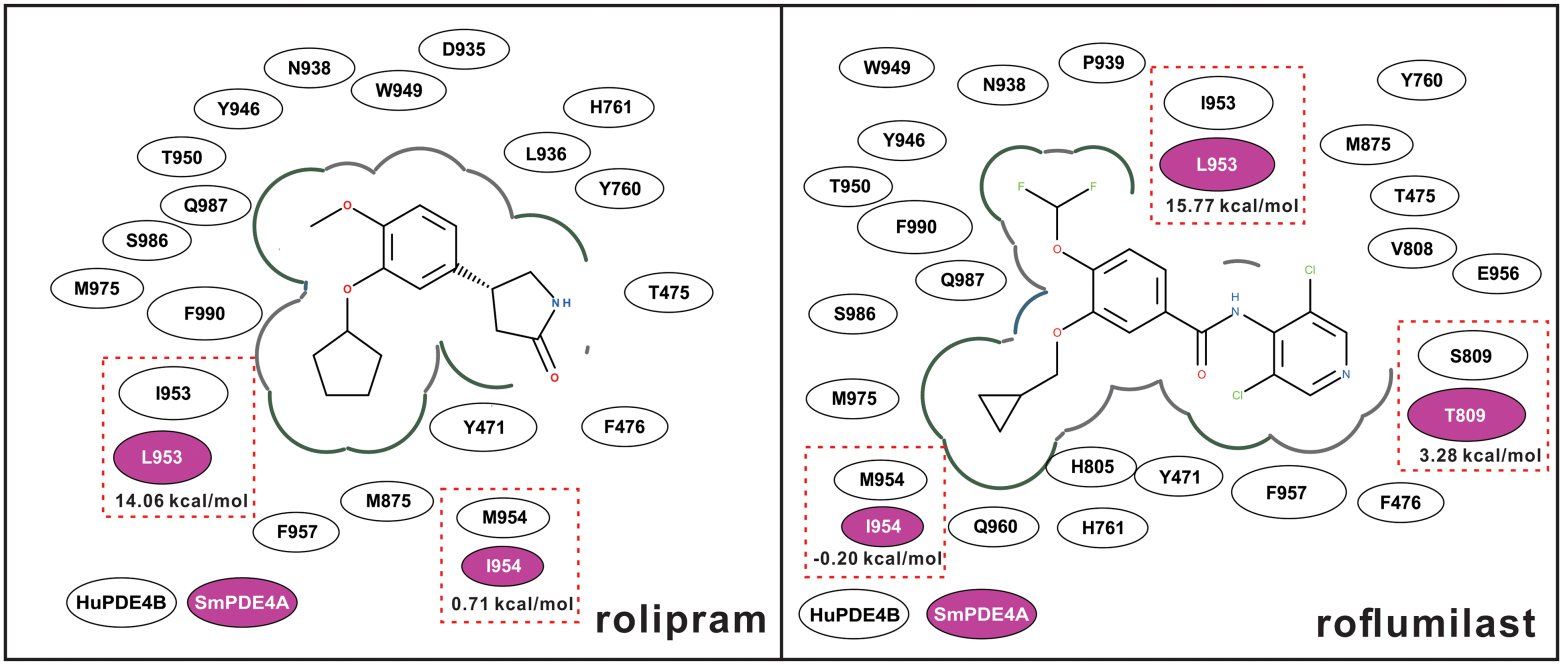
2D interaction diagram of rolipram and roflumilast with huPDE4B and SmPDE4A. Molecular models of each enzyme in complex with rolipram and roflumilast were built using ICM-pro and huPDE4B1 as a template (PDB ID: 4X0F) [79]. The amino acid residues in the huPDE4B1 and SmPDE4 binding sites that interact directly with the ligands are shown as ovals. Those that distinguish the schistosome ortholog are shown in magenta and the consequent changes in binding free energies are indicated underneath. The residue numbers are consistent with the alignment presented in Fig 2.

Fig 4 shows that the inhibition by the PDE4 catechol inhibitors, rolipram and roflumilast, is approximately 20-fold weaker for the schistosome enzyme compared to the human ortholog. To interpret these data, molecular models of each enzyme in complex with rolipram and roflumilast were built using ICM-pro and huPDE4B1 as a template (PDB ID: 4X0F) [79]. The ligand-protein interaction diagrams of rolipram and roflumilast are shown in Fig 8. The ligand-binding residues are highly conserved between both enzymes with the exception of two and three differences for binding to rolipram and roflumilast, respectively. Specifically, for rolipram-binding, I953 and M954 in huPDE4 are replaced by L and I in SmPDE4A, respectively (Fig 8, left panel). For binding to roflumilast, the same residues are changed in the same manner with the addition of a S→T809 substitution (Fig 8, right panel). Notably, and common for both inhibitors, the switch from I953 to L953 would make ligand binding unfavorable as shown by the high positive changes of binding free energies (14.06 kcal/mol and 15.77 kcal/mol for rolipram and roflumilast, respectively). This change helps explain the weaker inhibition measured for SmPDE4A with rolipram and roflumilast compared to the human enzyme.

## Discussion

A number of benzoxaboroles have been successfully brought through to the clinic and/or market for a variety of molecular drug targets, including aminoacyl-tRNA synthetase [70] and huPDE4 [73], and disease conditions [74, 75], such as Human African Trypanosomiasis [76]. Accordingly, we leveraged a benzoxaborole library from Anacor Pharmaceuticals to identify new drug development opportunities for schistosomiasis, a disease for which treatment currently relies on just one drug, praziquantel.

We first employed a phenotypic screen of 1,085 benzoxaboroles using *S*. *mansoni* somules to identify phenotypes of interest. We resolved three phenotype response groups: (i) 104 compounds eliciting an early and sustained hypermotile phenotype, including 30% that also induced degenerative changes; (ii) 94 compounds that yielded a range of phenotypic responses and (iii) 887 compounds that yielded no phenotype. The possibility that a PDE4 may be a target of interest arose via a statistically significant association for compounds that induced sustained hypermotility in the parasite and were sub-micromolar inhibitors of huPDE4. This notion was strengthened by a previous report that a mutation (*ce268*) of the single *pde4* gene in *C*. *elegans* causes hypermotility [103]. Given the circumstantial evidence, therefore, we searched for and identified four PDE4-like gene sequences in the *S*. *mansoni* genome, which we termed SmPDE4A through SmPDE4D.

Of the four putative PDE4 proteins identified in the *S*. *mansoni* genome, SmPDE4A is the most similar in protein sequence and inferred domain architecture to huPDE4 whereas the other three are more divergent in various respects such as possessing an extended N-terminal domain (SmPDE4B), sequence inserts (SmPDE4B and D) or sequence truncations (SmPDE4C). At this time, the possible functional significance of the sequence variations is unclear, however, based on the conservation of key amino acids directly involved in catalysis, all four gene products may be active. For each of the four protein sequences, corresponding expression products were identified in various developmental stages of *S*. *mansoni* relevant to infection in humans and orthologs of three of the SmPDE4 sequences (A, B and D) were identified in adults and/or somules of *S*. *haematobium* and *S*. *japonicum*.

Because of SmPDE4A’s greater similarity in sequence and domain organization to huPDE4, which had already been functionally expressed in bacteria during a campaign to develop benzoxaborole inhibitors [74], we chose to recombinantly express this enzyme and determine whether, for exemplar benzoxaboroles, an association between enzyme inhibition and phenotypic effects on the parasite existed. After expression of the SmPDE4A catalytic domain in *E*. *coli* and chromatographic purification, an enzyme that was catalytically active against the relevant cAMP substrate was obtained.

For seven exemplar benzoxaboroles, the potency of inhibition of SmPDE4A trended with the severity of parasite hypermotility, either recorded observationally in somules and adults, or using Wormassay, an image-based method to measure motility in adults [102]. For the most potent benzoxaborole inhibitors (**1–4**) of SmPDE4A, somules and adults underwent degenerative changes in addition to, and perhaps, as a consequence of, the extreme hypermotility recorded. Neither the sustained hypermotility nor degeneracy was noted for the weaker inhibitors of SmPDE4A. Although we cannot discount the possibility that the inhibitors tested interact with one or more of the other three putative PDE4s identified in *S*. *mansoni*, or indeed, other phosphodiesterases, the trends uncovered for the benzoxaboroles tested would indicate that the induction of parasite hypermotility and degeneracy is, at the least, mediated via inhibition of SmPDE4A, an interpretation that is consistent with the data for *C*. *elegans* transfected with a cDNA for SmPDE4A (discussed below). Relevant in this context is that the 5-(3-cyanopyridyl-6-oxy) benzoxaborole scaffold represented in the most potent SmPDE4A inhibitors (Fig 4) provides between 4 and >2,000-fold better potency (IC_50_ values) for huPDE4 over other huPDE enzymes [73].

The catechol, roflumilast, although a potent inhibitor of SmPDE4A, produced only marginal and transient phenotypic effects on the parasite. The reason(s) for this is unclear, but may be due to a lack of penetrance or rapid metabolism of the catechol by the parasite. The second catechol tested, rolipram, was inactive against SmPDE4 and, again, marginally bioactive. Both catechols are considerably weaker inhibitors of SmPDE4A than huPDE4 (approximately 20-fold). Molecular modeling revealed that there are up to three amino acid residue differences in the ligand-binding sites between SmPDE4A and huPDE4, but that the change at one position in particular, namely I→L953, would generate an unfavorable binding free energy value that would contribute to the weaker inhibition values measured for SmPDE4A with the catechols. Given how similar the ligand binding sites are otherwise, the differences noted could be important in a future campaign to derive more parasite-specific inhibitors and decrease potential off-target interactions with host PDE4.

To determine whether SmPDE4A can operate as a *bona fide* PDE4 in a heterologous biological system, we generated two *C*. *elegans* transgenic lines for full-length *smpde4a* on the *ce268* background, which lacks a functional *pde4* gene and is, consequently, hypermotile relative to WT worms [103]. The decision to use *C*. *elegans* as a functional read-out was motivated by the fact that genetic manipulation of *S*. *mansoni*, in spite of progress in this area [78], is still not a standardized undertaking. Both *smpde4a* transgene lines depressed hypermotility in *ce268* worms to the levels measured for WT–an original demonstration that a platyhelminth gene can compensate for gene functionality in this nematode model. The finding opens the possibility of using the *smpde4a* transgenic *C*. *elegans* as a research tool to perform mutational/mechanistic studies on enzyme function.

To understand whether the transgenic *C*. *elegans* system responded to PDE4 inhibitors, we tested the two catechols, rolipram and roflumilast, and two benzoxaboroles, compounds **2** and **4**, with the *pde4(ce268);smpde4* lines. Encouragingly, all of the inhibitors tested increased worm motility demonstrating that they engage and inhibit the SmPDE4A transgene. This raises the interesting possibility that the current transgenic model could be a useful tool in the further development of more specific inhibitors (see below), especially considering that the bacterial expression of ‘long isoform’ huPDE4, *i*.*e*., including both UCR1 and UCR2, is associated with difficulties relating to activity and the aggregation of different molar forms [79].

The finding that rolipram produced a modest, yet statistically significant, increase in the motility of transgenic *C*. *elegans* was initially surprising given the compound’s apparent lack of inhibition of the recombinant catalytic domain of SmPDE4A (IC_50_ >10 μM). One possible explanation may lie in the recent demonstration (consistent with earlier reports [82, 83, 104, 105]) that huPDE4B dimerizes via certain residues in UCR1 and UCR2, and that the UCR2 from one monomer contributes to the topography of the active site of the other monomer [79] (Fig 2). This UCR2-mediated alteration of the active site increases the rolipram-binding contacts and accounts for the existence of a high-affinity inhibition of huPDE4 by rolipram versus a low-affinity inhibition that involves the catalytic domain only [97, 98, 106]. In support of this explanation, the residues in UCR1 and UCR2 that contribute to the dimerization interface in huPDE4 are strongly conserved in SmPDE4A (and isoforms B and C, but not D; Fig 2). Also, the rolipram-facing residue in the UCR2 of huPDE4B (Y471 in Fig 2 designated Y274 in [79]) that enhances the binding potential of rolipram is conserved in SmPDE4A (and isoform B, but not C and D). Thus, it is conceivable that the UCR2 region present in the full length SmPDE4A cDNA that was transfected into the *C*. *elegans pde4(ce268)* mutant provides the additional necessary contacts for rolipram’s enhanced binding and induction of hypermotility. This interaction would, however, imply adjustments in the pose and contacts made by rolipram in the SmPDE4A binding site given the unfavorable binding presence of L953 compared to I953 in huPDE4. Unfortunately, our attempts to perform the corollary experiment of transfecting *C*. *elegans* with a truncated form of SmPDE4, *i*.*e*., minus the UCR2 domain, and provide support for UCR2’s contribution to rolipram’s enhanced binding were unsuccessful. If confirmed, however, then the extra specificity determinants present in the UCR2-augmented binding site (in addition to other control elements in the full-length enzyme [79, 107]) could be exploited in a program to improve inhibitor specificity especially given the strong similarities between the ligand binding sites of SmPDE4A and huPDE4 noted above.

To conclude, a phenotypic screen of a benzoxaborole collection with *S*. *mansoni* identified a particular phenotype-chemotype association that suggested an underlying PDE4 molecular target. An association between inhibition of the recombinant SmPDE4A, and parasite hypermotility and degeneration was noted. Employing *C*. *elegans* as a transgene expression system, we confirmed SmPDE4A’s contribution to modulating worm motility and its relevance as the molecular target for benzoxaborole inhibitors. The applicability of *C*. *elegans* as screening platform for small molecules to flatworm (schistosome) molecular targets, coupled with the differences noted between the human and schistosome PDE4s could support a structure-based approach to optimize inhibitor specificity, bioavailability and safety.

## Methods

### Ethics statement

Maintenance and handling of vertebrate animals were carried out in accordance with a protocol (AN107779) approved by the Institutional Animal Care and Use Committee at the University of California San Francisco. UCSF-IACUC derives its authority from the United States Public Health Service Policy on Humane Care and Use of Laboratory Animals, and the Animal Welfare Act and Regulations.

### *S*. *mansoni* life-cycle

We employ a Puerto Rican isolate of *S*. *mansoni* that is cycled between *Biomphalaria glabrata* snails and female Golden Syrian hamsters (infected at 4–6 weeks of age) as intermediate and definitive hosts, respectively. The acquisition, preparation and *in vitro* maintenance of mechanically transformed somules (derived from infective stage cercariae) and adults have been described [99, 108].

### Cloning and expression of SmPDE4

The catalytic domain of SmPDE4A (Smp_134140; XM_002573613; residues 668–1,060 in Fig 2) was synthesized (Genscript) with codons optimized for *Escherichia coli* expression (including a translation-start methionine codon) and cloned into the pET15b vector to yield an N-terminally His_6_-tagged protein. The protein was produced in *E*. *coli* BL21(DE3) cells grown in Terrific Broth medium supplemented with 0.1 mM zinc acetate and 50 μg/ml carbenicillin. For the large scale expression of recombinant SmPDE4A, cells were grown at 37°C to an OD_600_ approaching 0.5, the temperature was then dropped to 15°C, and the cells induced for 24 h with 0.1 mM isopropyl β-D-1-thiogalactopyranoside. Cells were collected by centrifugation at 4°C, flash frozen in liquid nitrogen and stored at -80°C.

For purification, frozen cells were suspended (1 g/ 5 ml) in 20 mM TRIS-HCl, pH 7.2, 250 mM NaCl, 10 mM imidazole, 1 mM phenyl methane sulfonyl fluoride (PMSF), and once fully homogenous, were lysed by microfluidization. Cellular debris was centrifuged at 4°C for 1 h at 12,500 *g*. The resulting lysate was then purified by metal-ion affinity chromatography using a His-TRAP FF column (GE Healthcare). Prior to purification, the column was washed with 10 column volumes of elution buffer (20 mM TRIS-HCl, pH 7.2, 250 mM NaCl, 500 mM imidazole) and equilibrated with 10 column volumes of binding buffer (20 mM TRIS-HCl, pH 7.2, 250 mM NaCl, 10 mM imidazole). The protein eluted at ~27.5% elution buffer. Major fractions containing the protein of interest were combined, concentrated and treated with an equal volume of 20 mM TRIS-HCl, pH 8.0, 2 M (NH_4_)_2_SO_4_. This was done in order to prepare the protein sample for hydrophobic interaction chromatography using a HiTRAP Butyl HP column (GE Healthcare). The column was equilibrated with binding buffer (20 mM TRIS-HCl, pH 8.0, 1 M (NH_4_)_2_SO_4_), followed by loading of SmPDE4A and elution with a linear gradient of 20 mM TRIS-HCl, pH 8.0. The protein eluted at ~50% elution buffer. Fractions containing the protein of interest were pooled, concentrated and buffer exchanged to decrease the concentration of (NH_4_)_2_SO_4_ to below 5 mM. As a final step, the protein was purified by ion-exchange chromatography using a Mono Q column (GE Healthcare). The column was equilibrated with binding buffer (20 mM TRIS-HCl, pH 8.0) followed by loading of SmPDE4A and elution with a linear gradient of 20 mM TRIS-HCl, pH 8.0, 1 M NaCl. The protein eluted at ~65% elution buffer. Fractions containing the protein of interest were pooled and concentrated, and the purity assessed by SDS-PAGE. The concentration of recombinant SmPDE4A was estimated by the Bradford Assay (BioRad) using bovine serum albumin (BSA) as a standard.

### PDE4 inhibition assay

Assay of PDE4 enzymatic activity was as described [96]: huPDE4B2 was purchased from Proteros Biostructures, GmbH, Martinsried, Germany. The reaction contained 0.15 μM [^3^H]-cAMP (10 uCi/ml; Perkin Elmer, Waltham, MA) and activity was measured by ZnSO_4_/Ba(OH)_2_ precipitation of the AMP product after reaction quenching. The precipitate was collected by filtration onto Multi-Screen HTS FB plates (Millipore, Billerica, MA), washed and then dried for quantitation of radioactivity. For tests with PDE4 inhibitors, fifty percent inhibitory concentration (IC_50_) values were calculated based on a four-parameter logistic equation: the means and number of replicates are reported in Fig 4. Racemate rolipram was purchased from Sigma (Cat. no. R6520) and roflumilast was from Selleckchem (cat. no. S2131).

### Phenotypic screens with *S*. *mansoni* somules and adults *in vitro*

Phenotypic screens involving somules and adults were carried out as described [99–101, 109]. For somules, approximately 300 parasites, newly transformed from cercariae [110], were manually dispensed into flat-bottomed 96-well plates (Corning Inc., cat. # 3599) containing 100 μl Basch medium and 4% FBS [99, 111]. Compound was then added in a volume of 1 μl DMSO and the final volume brought up to 200 μl with medium. The final compound concentration was 5 μM; somules were incubated for 6 days at 37°C under 5% CO_2_.

Adult schistosome screens were performed in 24-well plates (Corning Inc., cat. # 3544) using five worm pairs per well in a final volume of 2 ml of the above Basch medium. Compound was added in a volume of 1 μl DMSO such that the final concentration was 10 μM. Incubations were maintained for 3 days at 37°C under 5% CO_2_.

Parasite responses to chemical insult were adjudicated visually every 24 h (also at the 1 h time point for adults) using an inverted microscope and employed a constrained nomenclature of phenotype descriptors (*e*.*g*., rounding, degeneration, overactivity and slowed motility) as described [99–101]. For adult parasites, in addition to observation-based annotations, we employed Wormassay [102] to measure worm motility. Briefly, Wormassay comprises a commodity digital movie camera connected to an Apple personal computer that operates an open source software application to automatically process multiple wells (in 6-, 12- or 24-well plate formats). The application detects worm-induced changes in the occupation and vacancy of pixels between frames (outputted as an average ± S.D.). Worm motion was quantified using the “Consensus Voting Luminance Difference” option.

### Sequence-analysis and expression-profiling of SmPDE4 and its orthologs

To determine in which developmental stages the SmPDE4 genes are expressed, the GeneDB (http://www.genedb.org/Homepage) Gene IDs for SmPDE4A (Smp_134140), SmPDE4B (Smp_141980), SmPDE4C (Smp_129270) and SmPDE4D (Smp_044060) were each used as key words to search for the respective sequences. The “Transcript Expression” file was selected for each sequence to view transcriptomic expression data [65, 66]. To determine whether the SmPDE4 genes are expressed differentially in adult male and female parasites, the amino acid sequences were queried via tBLASTn in NCBI (http://ncbi.nlm.nih.gov/) against the EST (Expressed Sequence Tag) database and constraining the organism ID to “*Schistosoma*” (taxid: 6181). Only the information associated with returned sequences that shared ≥ 97% identity with the query sequence was scrutinized.

To identify orthologous sequences in the genomes of the human, *C*. *elegans*, *S*. *haematobium* [94] and *S*. *japonicum* [95], each SmPDE4 protein sequence was analyzed via tBLASTn in NCBI, again constraining for the taxid ID of 6181. The returned sequences that shared an identity of 30% or more were subsequently analyzed via BLASTp (at NCBI and GeneDB) to (i) obtain the full length sequence (ii) confirm the accession (gene ID) numbers and (iii) determine the sequence identity. Then, a sequence alignment was generated using the PRABI (Pôle Rhône-Alpes de Bioinformatique) MULTALIN tool (https://npsa-prabi.ibcp.fr/) to define the relative positions of the various PDE4 domains (UCR1, UCR2 and the catalytic domain). The catalytic domains were then used as queries via BLASTp in NCBI to determine sequence identities with the other orthologs.

### Molecular modeling of SmPDE4

Modeling was performed with the internal coordinate mechanics (ICM-pro) package for structure prediction, homology modeling and docking [112]. HuPDE4B1 in complex with (R)-(-)-rolipram (PDB code 4X0F; [79]) was used to build models incorporating the rolipram binding site. The residues surrounding the binding site of rolipram were mutated into the corresponding residues in SmPDE4A to build a SmPDE4A-rolipram model. The residue side chains around the binding site of rolipram in the human and parasite enzymes were then globally optimized using Biased Probability Monte Carlo (BPMS) sampling [112] with ICM energy functions in the context of rolipram. Similarly, the model of huPDE4B-roflumilast was built based on the PDB structures 4X0F [79] and 1XOQ [113]. A model of SmPDE4A-roflumilast was built by the mutating residues around the binding site of roflumilast and globally optimized with BPMS sampling in the context of roflumilast.

### Ligand-residue interaction diagram and *in silico* mutation analysis

With the models for huPDE4B1 and SmPDE4A in complex with rolipram and roflumilast, 2D diagrams of the ligand-residue interactions were built using the requisite tool in ICM-pro. The hydrophobic interaction cutoff was 5.0 Å. Then, with the model of huPDE4B1 in complex with rolipram and roflumilast, the residues around the binding site of rolipram and roflumilast that distinguish SmPDE4A from the human ortholog were mutated. The differences in ligand binding free energies were calculated using following equations:

$$\Delta \Delta G_{bind}=\Delta G_{bind}^{mut}-\Delta G_{bind}^{wt}$$

$$\Delta G_{bind}=\left(E_{intra}^{comp}-E_{intra}^{parts}\right)+\left(\Delta G_{solv}^{comp}-\Delta G_{solv}^{parts}\right)$$

where $\Delta G_{bind}^{wt}$ represents the binding free energy of protein and ligand in WT huPDE4B1; $\Delta G_{bind}^{mut}$ represents the binding free energy of protein and ligand in those residues mutated in huPDE4B1; $E_{intra}^{comp}$ represents the internal energy of the protein-ligand complex and $E_{intra}^{parts}$ represents the sum of the internal energy of protein and ligand. Similarly, $\Delta G_{solv}^{comp}$ represents the solvation energy of the protein-ligand complex and $\Delta G_{solv}^{parts}$ represents the sum of solvation energies of protein and ligand [114]

### *C*. *elegans* strains and culture methods

All strains were cultured at 20°C on nematode growth medium (NGM) plates seeded with *E*. *coli* strain OP50 [115]. N2 Bristol was used as the WT reference strain. The mutant strain used in this study, *pde4(ce268)*, carries a D448N mutation relative to WT *pde4* and is encoded by the *C*. *elegans* gene denoted as R153.1. The D448N change disrupts the catalytic domain by changing one of the four active residues that together chelate an active-site zinc atom. The consequence is a strong decrease in gene function [103].

### Plasmid constructs and generation of *C*. *elegans* WT and pde4 transgenic mutants

Plasmids were constructed using Gateway Technology (Invitrogen) reagents as described [116]. The entire *S*. *mansoni* SmPDE4A sequence (Smp_134140; XM_002573613) was PCR amplified from the start codon to immediately preceding the stop codon (2–1880 bp) using mixed sex, adult *S*. *mansoni* cDNA. PCR primers contained the gateway *att*B recombining sequences (in lower case): SmPDE4Fw, 5’- ggggacaagtttgtacaaaaaagcaggctTGGAGTTACGAACCGATAAAGTGATTTCATC-3’ and SmPDE4Rv, 5’- ggggaccactttgtacaagaaagctgggTATGTGTTTCCTGAAGTTGTAGA. The PCR fragment was cloned into a donor vector pKA5 (pDONR-221) and the correct sequence confirmed. Then, the entry vector pKA5-SmPDE4A was recombined with the 2 kb of sequence upstream of the start site of *unc-119* promoter [117–119] into a Gateway destination vector containing GFP (pKA453) to obtain promoter::SmPDE4A::intercistronic::gfp polycistronic fusions as previously described [120]. This also allows for co-expression of GFP and SmPDE4A from the same transcript without modifying SmPDE4A and facilitates the selection of transgenic animals via the GFP tag.

To generate *C*. *elegans* that carry the SmPDE4A transgene, the plasmids described above were purified and microinjected into the gonads of both WT (N2) and *pde4(ce268)* mutant strains at a concentration of 50 ng/μl. The injected worms (P0) were transferred to individual freshly seeded bacteria plates and allowed to reproduce. F1 progeny were screened for evidence of transgene expression based on the GFP marker. Each transgenic F1 was then singled onto a new plate. This process was repeated until lines that stably transmit the transgene were established in WT or *pde(ce268)* animal backgrounds. To verify the consistency of GFP expression in the transgenic lines, 10–20 transgenic animals from each line were examined using a Zeiss Axioplan II stereoscope equipped with a FITC/GFP filter (emission 500–515 nm).

### *C*. *elegans* motility assays

Compounds were directly added onto the OP50 food source at a 100 μM final concentration or the equivalent 0.2% DMSO as control. Previously synchronized early L4 stage larvae were cultivated on NGM plates with OP50 and compounds at 20°C for 16 h. For each experimental condition and transgenic line, motility was measured for 10–20 animals. The animals were washed twice in S basal buffer, transferred onto a new NGM plate in the absence of bacteria. After a brief period of recovery from this manipulation, locomotion was measured by counting the number of body bends in 30 s intervals under a stereoscope.

## Supporting information

S1 Fig Multiple sequence alignment of schistosome PDE4A orthologs. The alignment was generated using the PRABI (Pôle Rhône-Alpes de Bioinformatique) MULTALIN tool (https://npsa-prabi.ibcp.fr/). The image formatting is as presented in Fig 2. The Upstream Conserved Regions, UCR1 and UCR 2, and catalytic domains are indicated in blue, green and pink, respectively. The linker regions, LR1 and LR2, and the predicted PKA phosphorylation site in UCR1 are indicated. The conserved PDE signature motif HNX_2_HNX_N_E/D/QX_10_HDX_2_HX_25_E is indicated with blue circles and those residues that coordinate directly with the catalytic zinc in the substrate binding pocket are also indicated by the red circles. The gene identifiers for the *S*. *mansoni*, *S*. *haematobium* and *S*. *japonicum* sequences are Smp_134140, XM_012943524.1 and Sjp_0072560, respectively.
(TIF)

S2 Fig Multiple sequence alignment of schistosome PDE4B orthologs. The alignment was generated using the PRABI (Pôle Rhône-Alpes de Bioinformatique) MULTALIN tool (https://npsa-prabi.ibcp.fr/). The image formatting is as presented in Fig 2. The Upstream Conserved Regions, UCR1 and UCR 2, and catalytic domains are indicated in blue, green and pink, respectively. The linker regions, LR1 and LR2, and the predicted PKA phosphorylation site in UCR1 are indicated. The conserved PDE signature motif HNX_2_HNX_N_E/D/QX_10_HDX_2_HX_25_E is indicated with blue circles and those residues that coordinate directly with the catalytic zinc in the substrate binding pocket are also indicated by the red circles. The gene identifiers for the *S*. *mansoni*, *S*. *haematobium* and *S*. *japonicum* sequences are Smp_141980, XM_012941682.1 and Sjp_0099480, respectively.
(TIF)

S3 Fig Sequence alignment of schistosome PDE4D orthologs. The alignment was generated using the PRABI (Pôle Rhône-Alpes de Bioinformatique) MULTALIN tool (https://npsa-prabi.ibcp.fr/). The image formatting is as presented in Fig 2. The Upstream Conserved Region, UCR 2, and the catalytic domain are indicated in green and pink, respectively: the linker region, LR2, is shown by the blue horizontal line. UCR1 and LR1 appear to be absent. The conserved PDE signature motif HNX_2_HNX_N_E/D/QX_10_HDX_2_HX_25_E is indicated with blue circles and those residues that coordinate directly with the catalytic zinc in the substrate binding pocket are also indicated by the red circles. The gene identifiers for the *S*. *mansoni* and *S*. *haematobium* sequences are Smp_044060 and XM_012937519.1, respectively.
(TIF)

S4 Fig Examples of adult *S*. *mansoni* after exposure to benzoxaborole compound 2. Parasites were incubated in the absence (**A**, **B**) or presence (**C-F**) of 10 μM compound **2** for 3 days as described in the text. The anterior of worms is leftmost in each panel. (**A, B**) Control worm pairs with males adhering to the well surface via the anterior oral and ventral suckers. The darker, thinner female is held within and is seen looping out from the male’s gynecophoral canal. (**C, D**) Female worms demonstrating bulging of the body wall (arrows) and a general derangement of body shape, particularly in **D**. Likewise, the male worms (**E**, **F**) have a deranged body shape: arrows in **E** point to areas of lifting (blebbing) of the tegument (surface). Bar = 150 μm.
(TIF)

S1 Table Developmental expression of PDE4 genes in *S*. *mansoni* and their orthologs in *Schistosoma haematobium* and *Schistosoma japonicum*. When information was not available regarding gene expression in a particular developmental stage, the corresponding cell was left blank. See text for details.
(DOCX)

S2 Table Percentage protein sequence identities between full length PDE4 orthologs. A SmPDE4C ortholog was not found in *S*. *haematobium*, and SmPDE4C and D orthologs were absent in *S*. *japonicum*.
(DOCX)

S3 Table Percentage protein sequence identities between the catalytic domains of PDE4 orthologs. A SmPDE4C ortholog was not found in *S*. *haematobium*, and SmPDE4C and D orthologs were absent in *S*. *japonicum*.
(DOCX)

## References

[1] Abdul-Ghani RA, Loutfy N, Hassan A. Experimentally promising antischistosomal drugs: a review of some drug candidates not reaching the clinical use. Parasitol Res. 2009;105(4):899–906. doi: 10.1007/s00436-009-1546-2 . 19588166 10.1007/s00436-009-1546-2

[2] Dőmling A, Khoury K. Praziquantel and schistosomiasis. ChemMedChem. 2010;5(9):1420–34. Epub 2010/08/03. doi: 10.1002/cmdc.201000202 . 20677314 10.1002/cmdc.201000202

[3] World Health Organization. Schistosomiasis: Fact sheet N°115: WHO; 2014. http://www.who.int/mediacentre/factsheets/fs115/en/.

[4] Caffrey CR. Schistosomiasis and its treatment. Future Med Chem. 2015;7(6):675–6. doi: 10.4155/fmc.15.27 . 25996057 10.4155/fmc.15.27

[5] Cioli D, Pica-Mattoccia L, Basso A, Guidi A. Schistosomiasis control: praziquantel forever? Mol Biochem Parasitol. 2014;195(1):23–9. Epub 2014/06/24. doi: 10.1016/j.molbiopara.2014.06.002 . 24955523 10.1016/j.molbiopara.2014.06.002

[6] Thetiot-Laurent SA, Boissier J, Robert A, Meunier B. Schistosomiasis chemotherapy. Angew Chem Int Ed Engl. 2013;52(31):7936–56. Epub 2013/07/03. doi: 10.1002/anie.201208390 . 23813602 10.1002/anie.201208390

[7] World Health Organization. Accelerating work to overcome the global impact of neglected tropical diseases–A roadmap for implementation 2012.

[8] London Declaration Stakeholders Working Group. 2014. http://unitingtocombatntds.org/resource/download-report.

[9] Aragon AD, Imani RA, Blackburn VR, Cupit PM, Melman SD, Goronga T, et al Towards an understanding of the mechanism of action of praziquantel. Mol Biochem Parasitol. 2009;164(1):57–65. doi: 10.1016/j.molbiopara.2008.11.007 . 19100294 10.1016/j.molbiopara.2008.11.007 PMC2886009

[10] Valentim CL, Cioli D, Chevalier FD, Cao X, Taylor AB, Holloway SP, et al Genetic and molecular basis of drug resistance and species-specific drug action in schistosome parasites. Science. 2013;342(6164):1385–9. Epub 2013/11/23. doi: 10.1126/science.1243106 . 24263136 10.1126/science.1243106 PMC4136436

[11] Wang W W L, Liang YS. Susceptibility or resistance of praziquantel in human schistosomiasis: a review. Parasitology Research. 2012;111(5):1871–7. doi: 10.1007/s00436-012-3151-z 23052781 10.1007/s00436-012-3151-z

[12] Andrews P, Thomas H, Pohlke R, Seubert J. Praziquantel. Med Res Rev. 1983;3(2):147–200. . 6408323 10.1002/med.2610030204

[13] Sabah AA, Fletcher C, Webbe G, Doenhoff MJ. *Schistosoma mansoni*: chemotherapy of infections of different ages. Exp Parasitol. 1986;61(3):294–303. . 3086114 10.1016/0014-4894(86)90184-0

[14] Xiao SH, Yue WJ, Yang YQ, You JQ. Susceptibility of *Schistosoma japonicum* to different developmental stages to praziquantel. Chin Med J (Engl). 1987;100(9):759–68. . 3127152

[15] Botros S, Pica-Mattoccia L, William S, El-Lakkani N, Cioli D. Effect of praziquantel on the immature stages of *Schistosoma haematobium*. Int J Parasitol. 2005;35(13):1453–7. doi: 10.1016/j.ijpara.2005.05.002 . 16002073 10.1016/j.ijpara.2005.05.002

[16] Caffrey CR, Secor WE. Schistosomiasis: from drug deployment to drug development. Curr Opin Infect Dis. 2011;24(5):410–7. Epub 2011/07/08. doi: 10.1097/QCO.0b013e328349156f . 21734570 10.1097/QCO.0b013e328349156f

[17] Doenhoff MJ, Cioli D, Utzinger J. Praziquantel: mechanisms of action, resistance and new derivatives for schistosomiasis. Curr Opin Infect Dis. 2008;21(6):659–67. Epub 2008/11/04. doi: 10.1097/QCO.0b013e328318978f . 18978535 10.1097/QCO.0b013e328318978f

[18] Bustinduy AL, Waterhouse D, de Sousa-Figueiredo JC, Roberts SA, Atuhaire A, Van Dam GJ, et al Population pharmacokinetics and pharmacodynamics of praziquantel in Ugandan children with intestinal schistosomiasis: higher dosages are required for maximal efficacy. mBio. 2016;7(4). doi: 10.1128/mBio.00227-16 . 27507822 10.1128/mBio.00227-16 PMC4992966

[19] Bühring KU, Diekman HW, Muller H, Garbe A, Nowak H. Metabolism of praziquantel in man. European Journal of Drug Metabolism and Pharmacokinetics. 1978;3:179–90.

[20] Olliaro P, Delgado-Romero P, Keiser J. The little we know about the pharmacokinetics and pharmacodynamics of praziquantel (racemate and R-enantiomer). J Antimicrob Chemother. 2014;69(4):863–70. doi: 10.1093/jac/dkt491 . 24390933 10.1093/jac/dkt491

[21] Meyer T, Sekljic H, Fuchs S, Bothe H, Schollmeyer D, Miculka C. Taste, a new incentive to switch to (R)-praziquantel in schistosomiasis treatment. PLoS Negl Trop Dis. 2009;3(1):e357 doi: 10.1371/journal.pntd.0000357 . 19159015 10.1371/journal.pntd.0000357 PMC2614124

[22] Beavo JA. Cyclic nucleotide phosphodiesterases: functional implications of multiple isoforms. Physiological reviews. 1995;75(4):725–48. . 7480160 10.1152/physrev.1995.75.4.725

[23] Maurice DH, Ke H, Ahmad F, Wang Y, Chung J, Manganiello VC. Advances in targeting cyclic nucleotide phosphodiesterases. Nat Rev Drug Discov. 2014;13(4):290–314. Epub 2014/04/02. doi: 10.1038/nrd4228 . 24687066 10.1038/nrd4228 PMC4155750

[24] Ahmad F, Murata T, Shimizu K, Degerman E, Maurice D, Manganiello V. Cyclic nucleotide phosphodiesterases: important signaling modulators and therapeutic targets. Oral Diseases. 2015;21(1):e25–50. doi: 10.1111/odi.12275 25056711 10.1111/odi.12275 PMC4275405

[25] Conti M, Beavo J. Biochemistry and physiology of cyclic nucleotide phosphodiesterases: essential components in cyclic nucleotide signaling. Annual Review of Biochemistry. 2007;76:481–511. doi: 10.1146/annurev.biochem.76.060305.150444 . 17376027 10.1146/annurev.biochem.76.060305.150444

[26] Kametani F, Haga S. Accumulation of carboxy-terminal fragments of APP increases phosphodiesterase 8B. Neurobiology of Aging. 2015;36(2):634–7. doi: 10.1016/j.neurobiolaging.2014.09.029 . 25457556 10.1016/j.neurobiolaging.2014.09.029

[27] Francis SH, Houslay MD, Conti M. Phosphodiesterase inhibitors: factors that influence potency, selectivity, and action. Handbook of Experimental Pharmacology. 2011;(204):47–84. doi: 10.1007/978-3-642-17969-3_2 . 21695635 10.1007/978-3-642-17969-3_2

[28] Keravis T, Lugnier C. Cyclic nucleotide phosphodiesterases (PDE) and peptide motifs. Curr Pharm Des. 2010;16(9):1114–25. . 20030615 10.2174/138161210790963760

[29] Omori K, Kotera J. Overview of PDEs and their regulation. Circulation Research. 2007;100(3):309–27. doi: 10.1161/01.RES.0000256354.95791.f1 . 17307970 10.1161/01.RES.0000256354.95791.f1

[30] Bender AT, Beavo JA. Cyclic nucleotide phosphodiesterases: molecular regulation to clinical use. Pharmacological Reviews. 2006;58(3):488–520. doi: 10.1124/pr.58.3.5 . 16968949 10.1124/pr.58.3.5

[31] Beavo JA, Brunton LL. Cyclic nucleotide research—still expanding after half a century. Nat Rev Mol Cell Biol. 2002;3(9):710–8. doi: 10.1038/nrm911 . 12209131 10.1038/nrm911

[32] Takemoto DJ, Hansen J, Takemoto LJ, Houslay MD. Peptide mapping of multiple forms of cyclic nucleotide phosphodiesterase. J Biol Chem. 1982;257(24):14597–9. . 6294071

[33] Houslay MD, Adams DR. PDE4 cAMP phosphodiesterases: modular enzymes that orchestrate signalling cross-talk, desensitization and compartmentalization. Biochem J. 2003;370(Pt 1):1–18. doi: 10.1042/BJ20021698 . 12444918 10.1042/BJ20021698 PMC1223165

[34] Houslay MD, Schafer P, Zhang KY. Keynote review: phosphodiesterase-4 as a therapeutic target. Drug Discov Today. 2005;10(22):1503–19. doi: 10.1016/S1359-6446(05)03622-6 . 16257373 10.1016/S1359-6446(05)03622-6

[35] Klussmann E. Protein-protein interactions of PDE4 family members—Functions, interactions and therapeutic value. Cell Signal. 2016;28(7):713–8. doi: 10.1016/j.cellsig.2015.10.005 . 26498857 10.1016/j.cellsig.2015.10.005

[36] Eskandari N, Bastan R, Peachell PT. Regulation of human skin mast cell histamine release by PDE inhibitors. Allergologia et Immunopathologia. 2015;43(1):37–41. doi: 10.1016/j.aller.2013.07.011 . 24231152 10.1016/j.aller.2013.07.011

[37] Gurney ME, D'Amato EC, Burgin AB. Phosphodiesterase-4 (PDE4) molecular pharmacology and Alzheimer's disease. Neurotherapeutics: The Journal of the American Society for Experimental NeuroTherapeutics. 2015;12(1):49–56. doi: 10.1007/s13311-014-0309-7 . 25371167 10.1007/s13311-014-0309-7 PMC4322084

[38] Azam MA, Tripuraneni NS. Selective Phosphodiesterase 4B Inhibitors: A Review. Scientia Pharmaceutica. 2014;82(3):453–81. doi: 10.3797/scipharm.1404-08 . 25853062 10.3797/scipharm.1404-08 PMC4318138

[39] Lipworth BJ. Phosphodiesterase-4 inhibitors for asthma and chronic obstructive pulmonary disease. Lancet. 2005;365(9454):167–75. doi: 10.1016/S0140-6736(05)17708-3 . 15639300 10.1016/S0140-6736(05)17708-3

[40] Zhang KY, Ibrahim PN, Gillette S, Bollag G. Phosphodiesterase-4 as a potential drug target. Expert Opin Ther Targets. 2005;9(6):1283–305. doi: 10.1517/14728222.9.6.1283 . 16300476 10.1517/14728222.9.6.1283

[41] Kumar N, Goldminz AM, Kim N, Gottlieb AB. Phosphodiesterase 4-targeted treatments for autoimmune diseases. BMC Medicine. 2013;11:96 doi: 10.1186/1741-7015-11-96 . 23557064 10.1186/1741-7015-11-96 PMC3616808

[42] Global Burden of Disease Study 2013 Collaborators. Global, regional, and national incidence, prevalence, and years lived with disability for 301 acute and chronic diseases and injuries in 188 countries, 1990–2013: a systematic analysis for the Global Burden of Disease Study 2013. Lancet. 2015;386(9995):743–800. Epub 2015/06/13. doi: 10.1016/S0140-6736(15)60692-4 . 26063472 10.1016/S0140-6736(15)60692-4 PMC4561509

[43] Fan Chung K. Phosphodiesterase inhibitors in airways disease. Eur J Pharmacol. 2006;533(1–3):110–7. doi: 10.1016/j.ejphar.2005.12.059 . 16458289 10.1016/j.ejphar.2005.12.059

[44] Heckman PR, Wouters C, Prickaerts J. Phosphodiesterase inhibitors as a target for cognition enhancement in aging and Alzheimer's disease: a translational overview. Curr Pharm Des. 2015;21(3):317–31. . 25159073 10.2174/1381612820666140826114601

[45] Cheng YF, Wang C, Lin HB, Li YF, Huang Y, Xu JP, et al Inhibition of phosphodiesterase-4 reverses memory deficits produced by Abeta25-35 or Abeta1-40 peptide in rats. Psychopharmacology. 2010;212(2):181–91. doi: 10.1007/s00213-010-1943-3 . 20640406 10.1007/s00213-010-1943-3

[46] Garcia-Osta A, Cuadrado-Tejedor M, Garcia-Barroso C, Oyarzabal J, Franco R. Phosphodiesterases as therapeutic targets for Alzheimer's disease. ACS Chemical Neuroscience. 2012;3(11):832–44. doi: 10.1021/cn3000907 . 23173065 10.1021/cn3000907 PMC3503343

[47] Akar F, Mutlu O, Komsuoglu Celikyurt I, Ulak G, Erden F, Bektas E, et al Zaprinast and rolipram enhances spatial and emotional memory in the elevated plus maze and passive avoidance tests and diminishes exploratory activity in naive mice. Medical Science Monitor Basic Research. 2014;20:105–11. doi: 10.12659/MSMBR.891149 . 25057848 10.12659/MSMBR.891149 PMC4117679

[48] Ricciarelli R, Fedele E. Phosphodiesterase 4D: an enzyme to remember. Br J Pharmacol. 2015;172(20):4785–9. doi: 10.1111/bph.13257 . 26211680 10.1111/bph.13257 PMC4621991

[49] Shakur Y, de Koning HP, Ke H, Kambayashi J, Seebeck T. Therapeutic potential of phosphodiesterase inhibitors in parasitic diseases. Handbook of Experimental Pharmacology. 2011;(204):487–510. doi: 10.1007/978-3-642-17969-3_20 . 21695653 10.1007/978-3-642-17969-3_20

[50] Wang C, Ashton TD, Gustafson A, Bland ND, Ochiana SO, Campbell RK, et al Synthesis and evaluation of human phosphodiesterases (PDE) 5 inhibitor analogs as trypanosomal PDE inhibitors. Part 1. Sildenafil analogs. Bioorg Med Chem Lett. 2012;22(7):2579–81. doi: 10.1016/j.bmcl.2012.01.119 . 22370268 10.1016/j.bmcl.2012.01.119 PMC3307826

[51] Ochiana SO, Gustafson A, Bland ND, Wang C, Russo MJ, Campbell RK, et al Synthesis and evaluation of human phosphodiesterases (PDE) 5 inhibitor analogs as trypanosomal PDE inhibitors. Part 2. Tadalafil analogs. Bioorg Med Chem Lett. 2012;22(7):2582–4. doi: 10.1016/j.bmcl.2012.01.118 . 22377518 10.1016/j.bmcl.2012.01.118 PMC3307956

[52] Berriman M, Ghedin E, Hertz-Fowler C, Blandin G, Renauld H, Bartholomeu DC, et al The genome of the African trypanosome *Trypanosoma brucei*. Science. 2005;309(5733):416–22. Epub 2005/07/16. doi: 10.1126/science.1112642 . 16020726 10.1126/science.1112642

[53] Bland ND, Wang C, Tallman C, Gustafson AE, Wang Z, Ashton TD, et al Pharmacological validation of *Trypanosoma brucei* phosphodiesterases B1 and B2 as druggable targets for African sleeping sickness. J Med Chem. 2011;54(23):8188–94. doi: 10.1021/jm201148s . 22023548 10.1021/jm201148s PMC3228873

[54] Oberholzer M, Marti G, Baresic M, Kunz S, Hemphill A, Seebeck T. The *Trypanosoma brucei* cAMP phosphodiesterases TbrPDEB1 and TbrPDEB2: flagellar enzymes that are essential for parasite virulence. FASEB J. 2007;21(3):720–31. doi: 10.1096/fj.06-6818com . 17167070 10.1096/fj.06-6818com

[55] Ochiana SO, Bland ND, Settimo L, Campbell RK, Pollastri MP. Repurposing human PDE4 inhibitors for neglected tropical diseases. Evaluation of analogs of the human PDE4 inhibitor GSK-256066 as inhibitors of PDEB1 of *Trypanosoma brucei*. Chem Biol Drug Des. 2015;85(5):549–64. doi: 10.1111/cbdd.12443 . 25283372 10.1111/cbdd.12443 PMC4385514

[56] Rascon A, Soderling SH, Schaefer JB, Beavo JA. Cloning and characterization of a cAMP-specific phosphodiesterase (TbPDE2B) from *Trypanosoma brucei*. Proc Natl Acad Sci U S A. 2002;99(7):4714–9. doi: 10.1073/pnas.002031599 . 11930017 10.1073/pnas.002031599 PMC123713

[57] de Koning HP, Gould MK, Sterk GJ, Tenor H, Kunz S, Luginbuehl E, et al Pharmacological validation of *Trypanosoma brucei* phosphodiesterases as novel drug targets. J Infect Dis. 2012;206(2):229–37. Epub 2012/02/01. doi: 10.1093/infdis/jir857 . 22291195 10.1093/infdis/jir857 PMC3379837

[58] Veerman J, van den Bergh T, Orrling KM, Jansen C, Cos P, Maes L, et al Synthesis and evaluation of analogs of the phenylpyridazinone NPD-001 as potent trypanosomal TbrPDEB1 phosphodiesterase inhibitors and in vitro trypanocidals. Bioorg Med Chem. 2016;24(7):1573–81. Epub 2016/03/05. doi: 10.1016/j.bmc.2016.02.032 . 26935942 10.1016/j.bmc.2016.02.032

[59] Orrling KM, Jansen C, Vu XL, Balmer V, Bregy P, Shanmugham A, et al Catechol pyrazolinones as trypanocidals: fragment-based design, synthesis, and pharmacological evaluation of nanomolar inhibitors of trypanosomal phosphodiesterase B1. J Med Chem. 2012;55(20):8745–56. Epub 2012/09/12. doi: 10.1021/jm301059b . 22963052 10.1021/jm301059b

[60] Johner A, Kunz S, Linder M, Shakur Y, Seebeck T. Cyclic nucleotide specific phosphodiesterases of *Leishmania major*. BMC Microbiology. 2006;6:25 doi: 10.1186/1471-2180-6-25 . 16522215 10.1186/1471-2180-6-25 PMC1431542

[61] Seebeck T, Sterk GJ, Ke H. Phosphodiesterase inhibitors as a new generation of antiprotozoan drugs: exploiting the benefit of enzymes that are highly conserved between host and parasite. Future Med Chem. 2011;3(10):1289–306. doi: 10.4155/fmc.11.77 . 21859303 10.4155/fmc.11.77 PMC3164761

[62] Alonso GD, Schoijet AC, Torres HN, Flawia MM. TcPDE4, a novel membrane-associated cAMP-specific phosphodiesterase from *Trypanosoma cruzi*. Mol Biochem Parasitol. 2006;145(1):40–9. doi: 10.1016/j.molbiopara.2005.09.005 . 16225937 10.1016/j.molbiopara.2005.09.005

[63] Yuasa K, Mi-Ichi F, Kobayashi T, Yamanouchi M, Kotera J, Kita K, et al PfPDE1, a novel cGMP-specific phosphodiesterase from the human malaria parasite *Plasmodium falciparum*. Biochem J. 2005;392(Pt 1):221–9. doi: 10.1042/BJ20050425 . 16038615 10.1042/BJ20050425 PMC1317681

[64] Howard BL, Harvey KL, Stewart RJ, Azevedo MF, Crabb BS, Jennings IG, et al Identification of potent phosphodiesterase inhibitors that demonstrate cyclic nucleotide-dependent functions in apicomplexan parasites. ACS Chem Biol. 2015;10(4):1145–54. doi: 10.1021/cb501004q . 25555060 10.1021/cb501004q

[65] Protasio AV, Tsai IJ, Babbage A, Nichol S, Hunt M, Aslett MA, et al A systematically improved high quality genome and transcriptome of the human blood fluke *Schistosoma mansoni*. PLoS Negl Trop Dis. 2012;6(1):e1455 Epub 2012/01/19. doi: 10.1371/journal.pntd.0001455 . 22253936 10.1371/journal.pntd.0001455 PMC3254664

[66] Berriman M, Haas BJ, LoVerde PT, Wilson RA, Dillon GP, Cerqueira GC, et al The genome of the blood fluke *Schistosoma mansoni*. Nature. 2009;460(7253):352–8. Epub 2009/07/17. nature08160 [pii] doi: 10.1038/nature08160 . 19606141 10.1038/nature08160 PMC2756445

[67] Matsuyama H, Takahashi H, Watanabe K, Fujimaki Y, Aoki Y. The involvement of cyclic adenosine monophosphate in the control of schistosome miracidium cilia. J Parasitol. 2004;90(1):8–14. Epub 2004/03/26. doi: 10.1645/GE-52R1 . 15040661 10.1645/GE-52R1

[68] Kawamoto F, Shozawa A, Kumada N, Kojima K. Possible roles of cAMP and Ca2+ in the regulation of miracidial transformation in *Schistosoma mansoni*. Parasitol Res. 1989;75(5):368–74. Epub 1989/01/01. . 2542928 10.1007/BF00931132

[69] Taft AS, Norante FA, Yoshino TP. The identification of inhibitors of *Schistosoma mansoni* miracidial transformation by incorporating a medium-throughput small-molecule screen. Exp Parasitol. 2010;125(2):84–94. Epub 2010/01/12. doi: 10.1016/j.exppara.2009.12.021 . 20060828 10.1016/j.exppara.2009.12.021 PMC2859107

[70] Rock FL, Mao W, Yaremchuk A, Tukalo M, Crepin T, Zhou H, et al An antifungal agent inhibits an aminoacyl-tRNA synthetase by trapping tRNA in the editing site. Science. 2007;316(5832):1759–61. Epub 2007/06/26. doi: 10.1126/science.1142189 . 17588934 10.1126/science.1142189

[71] Del Rosso JQ, Plattner JJ. From the test tube to the treatment room: fundamentals of boron-containing compounds and their relevance to dermatology. The Journal of Clinical and Aesthetic Dermatology. 2014;7(2):13–21. Epub 2014/03/01. . 24578778 PMC3935647

[72] Hernandez V, Crepin T, Palencia A, Cusack S, Akama T, Baker SJ, et al Discovery of a novel class of boron-based antibacterials with activity against gram-negative bacteria. Antimicrob Agents Chemother. 2013;57(3):1394–403. Epub 2013/01/09. doi: 10.1128/AAC.02058-12 . 23295920 10.1128/AAC.02058-12 PMC3591879

[73] Freund YR, Akama T, Alley MR, Antunes J, Dong C, Jarnagin K, et al Boron-based phosphodiesterase inhibitors show novel binding of boron to PDE4 bimetal center. FEBS Lett. 2012;586(19):3410–4. Epub 2012/07/31. doi: 10.1016/j.febslet.2012.07.058 . 22841723 10.1016/j.febslet.2012.07.058

[74] Jarnagin K, Chanda S, Coronado D, Ciaravino V, Zane LT, Guttman-Yassky E, et al Crisaborole Topical Ointment, 2%: A nonsteroidal, topical, anti-inflammatory phosphodiesterase 4 inhibitor in clinical development for the treatment of atopic dermatitis. Journal of Drugs in Dermatology: JDD. 2016;15(4):390–6. Epub 2016/04/07. . 27050693

[75] Paller AS, Tom WL, Lebwohl MG, Blumenthal RL, Boguniewicz M, Call RS, et al Efficacy and safety of crisaborole ointment, a novel, nonsteroidal phosphodiesterase 4 (PDE4) inhibitor for the topical treatment of atopic dermatitis (AD) in children and adults. Journal of the American Academy of Dermatology. 2016;75(3):494–503 e4. Epub 2016/07/16. doi: 10.1016/j.jaad.2016.05.046 . 27417017 10.1016/j.jaad.2016.05.046

[76] Jacobs RT, Plattner JJ, Nare B, Wring SA, Chen D, Freund Y, et al Benzoxaboroles: a new class of potential drugs for human African trypanosomiasis. Future Med Chem. 2011;3(10):1259–78. Epub 2011/08/24. doi: 10.4155/fmc.11.80 . 21859301 10.4155/fmc.11.80

[77] Drugs for Neglected Diseases initiative. SCYX-7158 Oxaborole: Drugs for Neglected Diseases initiative; 2017. https://www.dndi.org/diseases-projects/portfolio/scyx-7158/.

[78] Hagen J, Scheerlinck JP, Gasser RB. Knocking down schistosomes—promise for lentiviral transduction in parasites. Trends Parasitol. 2015;31(7):324–32. Epub 2015/05/03. doi: 10.1016/j.pt.2015.03.009 . 25933926 10.1016/j.pt.2015.03.009

[79] Cedervall P, Aulabaugh A, Geoghegan KF, McLellan TJ, Pandit J. Engineered stabilization and structural analysis of the autoinhibited conformation of PDE4. Proc Natl Acad Sci U S A. 2015;112(12):E1414–22. doi: 10.1073/pnas.1419906112 . 25775568 10.1073/pnas.1419906112 PMC4378417

[80] Houslay MD, Sullivan M, Bolger GB. The multienzyme PDE4 cyclic adenosine monophosphate-specific phosphodiesterase family: intracellular targeting, regulation, and selective inhibition by compounds exerting anti-inflammatory and antidepressant actions. Advances in Pharmacology. 1998;44:225–342. . 9547887 10.1016/s1054-3589(08)60128-3

[81] Houslay MD. Underpinning compartmentalised cAMP signalling through targeted cAMP breakdown. Trends Biochem Sci. 2010;35(2):91–100. doi: 10.1016/j.tibs.2009.09.007 . 19864144 10.1016/j.tibs.2009.09.007

[82] Xie M, Blackman B, Scheitrum C, Mika D, Blanchard E, Lei T, et al The upstream conserved regions (UCRs) mediate homo- and hetero-oligomerization of type 4 cyclic nucleotide phosphodiesterases (PDE4s). Biochem J. 2014;459(3):539–50. doi: 10.1042/BJ20131681 . 24555506 10.1042/BJ20131681 PMC4315173

[83] Richter W, Conti M. Dimerization of the type 4 cAMP-specific phosphodiesterases is mediated by the upstream conserved regions (UCRs). J Biol Chem. 2002;277(43):40212–21. doi: 10.1074/jbc.M203585200 . 12177055 10.1074/jbc.M203585200

[84] Beavo J.A. H MD, Francis S.H. Cyclic nucleotide phosphodiesterase superfamily Cyclic nucleotide phosphodiesterases in health and disease: Boca Raton: CRC Press 2006 p. 3–17.

[85] Xu RX, Hassell AM, Vanderwall D, Lambert MH, Holmes WD, Luther MA, et al Atomic structure of PDE4: insights into phosphodiesterase mechanism and specificity. Science. 2000;288(5472):1822–5. . 10846163 10.1126/science.288.5472.1822

[86] Sette C, Conti M. Phosphorylation and activation of a cAMP-specific phosphodiesterase by the cAMP-dependent protein kinase. Involvement of serine 54 in the enzyme activation. J Biol Chem. 1996;271(28):16526–34. . 8663227 10.1074/jbc.271.28.16526

[87] Hoffmann R, Wilkinson IR, McCallum JF, Engels P, Houslay MD. cAMP-specific phosphodiesterase HSPDE4D3 mutants which mimic activation and changes in rolipram inhibition triggered by protein kinase A phosphorylation of Ser-54: generation of a molecular model. Biochem J. 1998;333 (Pt 1):139–49. . 9639573 10.1042/bj3330139 PMC1219566

[88] MacKenzie SJ, Baillie GS, McPhee I, MacKenzie C, Seamons R, McSorley T, et al Long PDE4 cAMP specific phosphodiesterases are activated by protein kinase A-mediated phosphorylation of a single serine residue in Upstream Conserved Region 1 (UCR1). Br J Pharmacol. 2002;136(3):421–33. doi: 10.1038/sj.bjp.0704743 . 12023945 10.1038/sj.bjp.0704743 PMC1573369

[89] Baillie GS, MacKenzie SJ, McPhee I, Houslay MD. Sub-family selective actions in the ability of Erk2 MAP kinase to phosphorylate and regulate the activity of PDE4 cyclic AMP-specific phosphodiesterases. Br J Pharmacol. 2000;131(4):811–9. doi: 10.1038/sj.bjp.0703636 . 11030732 10.1038/sj.bjp.0703636 PMC1572393

[90] Hill EV, Sheppard CL, Cheung YF, Gall I, Krause E, Houslay MD. Oxidative stress employs phosphatidyl inositol 3-kinase and ERK signalling pathways to activate cAMP phosphodiesterase-4D3 (PDE4D3) through multi-site phosphorylation at Ser239 and Ser579. Cell Signal. 2006;18(11):2056–69. doi: 10.1016/j.cellsig.2006.07.018 . 16973330 10.1016/j.cellsig.2006.07.018

[91] MacKenzie SJ, Baillie GS, McPhee I, Bolger GB, Houslay MD. ERK2 mitogen-activated protein kinase binding, phosphorylation, and regulation of the PDE4D cAMP-specific phosphodiesterases. The involvement of COOH-terminal docking sites and NH2-terminal UCR regions. J Biol Chem. 2000;275(22):16609–17. . 10828059 10.1074/jbc.275.22.16609

[92] Ding D, Zhao Y, Meng Q, Xie D, Nare B, Chen D, et al Discovery of novel benzoxaborole-based potent antitrypanosomal agents. ACS Medicinal Chemistry Letters. 2010;1(4):165–9. doi: 10.1021/ml100013s . 24900190 10.1021/ml100013s PMC4007846

[93] Aravind L, Koonin EV. The HD domain defines a new superfamily of metal-dependent phosphohydrolases. Trends Biochem Sci. 1998;23(12):469–72. Epub 1998/12/30. . 9868367 10.1016/s0968-0004(98)01293-6

[94] Young ND, Jex AR, Li B, Liu S, Yang L, Xiong Z, et al Whole-genome sequence of *Schistosoma haematobium*. Nat Genet. 2012;44(2):221–5. Epub 2012/01/17. doi: 10.1038/ng.1065 . 22246508 10.1038/ng.1065

[95] Liu F, Zhou Y, Wang ZQ, Lu G, Zheng H, Brindley PJ, et al The *Schistosoma japonicum* genome reveals features of host-parasite interplay. Nature. 2009;460(7253):345–51. Epub 2009/07/17. doi: 10.1038/nature08140 . 19606140 10.1038/nature08140 PMC3747554

[96] Saldou N, Obernolte R, Huber A, Baecker PA, Wilhelm R, Alvarez R, et al Comparison of recombinant human PDE4 isoforms: interaction with substrate and inhibitors. Cell Signal. 1998;10(6):427–40. Epub 1998/08/28. . 9720765 10.1016/s0898-6568(97)00169-1

[97] Huston E, Pooley L, Julien P, Scotland G, McPhee I, Sullivan M, et al The human cyclic AMP-specific phosphodiesterase PDE-46 (HSPDE4A4B) expressed in transfected COS7 cells occurs as both particulate and cytosolic species that exhibit distinct kinetics of inhibition by the antidepressant rolipram. J Biol Chem. 1996;271(49):31334–44. . 8940140 10.1074/jbc.271.49.31334

[98] Bolger G, Michaeli T, Martins T, St John T, Steiner B, Rodgers L, et al A family of human phosphodiesterases homologous to the dunce learning and memory gene product of D*rosophila melanogaster* are potential targets for antidepressant drugs. Mol Cell Biol. 1993;13(10):6558–71. . 8413254 10.1128/mcb.13.10.6558-6571.1993 PMC364715

[99] Abdulla MH, Ruelas DS, Wolff B, Snedecor J, Lim KC, Xu F, et al Drug discovery for schistosomiasis: hit and lead compounds identified in a library of known drugs by medium-throughput phenotypic screening. PLoS Negl Trop Dis. 2009;3(7):e478 Epub 2009/07/15. doi: 10.1371/journal.pntd.0000478 . 19597541 10.1371/journal.pntd.0000478 PMC2702839

[100] Glaser J, Schurigt U, Suzuki BM, Caffrey CR, Holzgrabe U. Anti-schistosomal activity of cinnamic acid esters: eugenyl and thymyl cinnamate induce cytoplasmic vacuoles and death in schistosomula of *Schistosoma mansoni*. Molecules. 2015;20(6):10873–83. Epub 2015/06/16. doi: 10.3390/molecules200610873 . 26076109 10.3390/molecules200610873 PMC6272620

[101] Long T, Neitz RJ, Beasley R, Kalyanaraman C, Suzuki BM, Jacobson MP, et al Structure-bioactivity relationship for benzimidazole thiophene inhibitors of polo-like kinase 1 (PLK1), a potential drug target in *Schistosoma mansoni*. PLoS Negl Trop Dis. 2016;10(1):e0004356 Epub 2016/01/12. doi: 10.1371/journal.pntd.0004356 . 26751972 10.1371/journal.pntd.0004356 PMC4709140

[102] Marcellino C, Gut J, Lim KC, Singh R, McKerrow J, Sakanari J. WormAssay: a novel computer application for whole-plate motion-based screening of macroscopic parasites. PLoS Negl Trop Dis. 2012;6(1):e1494 Epub 2012/02/04. doi: 10.1371/journal.pntd.0001494 . 22303493 10.1371/journal.pntd.0001494 PMC3269415

[103] Charlie NK, Thomure AM, Schade MA, Miller KG. The Dunce cAMP phosphodiesterase PDE-4 negatively regulates G alpha(s)-dependent and G alpha(s)-independent cAMP pools in the *Caenorhabditis elegans* synaptic signaling network. Genetics. 2006;173(1):111–30. Epub 2006/04/21. doi: 10.1534/genetics.105.054007 . 16624912 10.1534/genetics.105.054007 PMC1461419

[104] Richter W, Conti M. The oligomerization state determines regulatory properties and inhibitor sensitivity of type 4 cAMP-specific phosphodiesterases. J Biol Chem. 2004;279(29):30338–48. doi: 10.1074/jbc.M312687200 . 15131123 10.1074/jbc.M312687200

[105] Bolger GB, Dunlop AJ, Meng D, Day JP, Klussmann E, Baillie GS, et al Dimerization of cAMP phosphodiesterase-4 (PDE4) in living cells requires interfaces located in both the UCR1 and catalytic unit domains. Cell Signal. 2015;27(4):756–69. doi: 10.1016/j.cellsig.2014.12.009 . 25546709 10.1016/j.cellsig.2014.12.009 PMC4371794

[106] Owens RJ, Catterall C, Batty D, Jappy J, Russell A, Smith B, et al Human phosphodiesterase 4A: characterization of full-length and truncated enzymes expressed in COS cells. Biochem J. 1997;326 (Pt 1):53–60. . 9337850 10.1042/bj3260053 PMC1218636

[107] Gurney ME, Burgin AB, Magnusson OT, Stewart LJ. Small molecule allosteric modulators of phosphodiesterase 4. Handbook of Experimental Pharmacology. 2011;(204):167–92. Epub 2011/06/23. doi: 10.1007/978-3-642-17969-3_7 . 21695640 10.1007/978-3-642-17969-3_7

[108] Štefanić S, Dvořák J, Horn M, Braschi S, Sojka D, Ruelas DS, et al RNA interference in Schistosoma mansoni schistosomula: selectivity, sensitivity and operation for larger-scale screening. PLoS Negl Trop Dis. 2010;4(10):e850 Epub 2010/10/27. doi: 10.1371/journal.pntd.0000850 . 20976050 10.1371/journal.pntd.0000850 PMC2957409

[109] Beckmann S, Long T, Scheld C, Geyer R, Caffrey CR, Grevelding CG. Serum albumin and alpha-1 acid glycoprotein impede the killing of *Schistosoma mansoni* by the tyrosine kinase inhibitor Imatinib. International Journal for Parasitology Drugs and Drug Resistance. 2014;4(3):287–95. doi: 10.1016/j.ijpddr.2014.07.005 . 25516839 10.1016/j.ijpddr.2014.07.005 PMC4266805

[110] Colley DG, Wikel SK. *Schistosoma mansoni*: simplified method for the production of schistosomules. Exp Parasitol. 1974;35(1):44–51. . 4815018 10.1016/0014-4894(74)90005-8

[111] Basch PF. Cultivation of *Schistosoma mansoni in vitro*. I. Establishment of cultures from cercariae and development until pairing. J Parasitol. 1981;67(2):179–85. . 7241277

[112] Abagyan R, Totrov M. Biased probability Monte Carlo conformational searches and electrostatic calculations for peptides and proteins. J Mol Biol. 1994;235(3):983–1002. Epub 1994/01/21. doi: 10.1006/jmbi.1994.1052 . 8289329 10.1006/jmbi.1994.1052

[113] Card GL, England BP, Suzuki Y, Fong D, Powell B, Lee B, et al Structural basis for the activity of drugs that inhibit phosphodiesterases. Structure. 2004;12(12):2233–47. Epub 2004/12/04. doi: 10.1016/j.str.2004.10.004 . 15576036 10.1016/j.str.2004.10.004

[114] Bordner AJ, Abagyan RA. Large-scale prediction of protein geometry and stability changes for arbitrary single point mutations. Proteins. 2004;57(2):400–13. Epub 2004/09/02. doi: 10.1002/prot.20185 . 15340927 10.1002/prot.20185

[115] Brenner S. The genetics of *Caenorhabditis elegans*. Genetics. 1974;77(1):71–94. . 4366476 10.1093/genetics/77.1.71 PMC1213120

[116] Srinivasan S, Sadegh L, Elle IC, Christensen AG, Faergeman NJ, Ashrafi K. Serotonin regulates C. elegans fat and feeding through independent molecular mechanisms. Cell Metabolism. 2008;7(6):533–44. doi: 10.1016/j.cmet.2008.04.012 . 18522834 10.1016/j.cmet.2008.04.012 PMC2495008

[117] Maduro M, Pilgrim D. Identification and cloning of unc-119, a gene expressed in the *Caenorhabditis elegans* nervous system. Genetics. 1995;141(3):977–88. Epub 1995/11/01. . 8582641 10.1093/genetics/141.3.977 PMC1206859

[118] Lemieux GA, Cunningham KA, Lin L, Mayer F, Werb Z, Ashrafi K. Kynurenic acid is a nutritional cue that enables behavioral plasticity. Cell. 2015;160(1–2):119–31. Epub 2015/01/17. doi: 10.1016/j.cell.2014.12.028 . 25594177 10.1016/j.cell.2014.12.028 PMC4334586

[119] Cunningham KA, Bouagnon AD, Barros AG, Lin L, Malard L, Romano-Silva MA, et al Loss of a neural AMP-activated kinase mimics the effects of elevated serotonin on fat, movement, and hormonal secretions. PLoS Genetics. 2014;10(6):e1004394 Epub 2014/06/13. doi: 10.1371/journal.pgen.1004394 . 24921650 10.1371/journal.pgen.1004394 PMC4055570

[120] Lee BH, Ashrafi K. A TRPV channel modulates *C*. *elegans* neurosecretion, larval starvation survival, and adult lifespan. PLoS Genetics. 2008;4(10):e1000213 doi: 10.1371/journal.pgen.1000213 . 18846209 10.1371/journal.pgen.1000213 PMC2556084

