# Supplementary figures and images for: Phenotypic, chemical and functional characterization of cyclic nucleotide phosphodiesterase 4 (PDE4) as a potential anthelmintic drug target
Source: PLoS Negl Trop Dis. 2017 Jul 13;11(7):e0005680. doi: 10.1371/journal.pntd.0005680 (PMC5526615; doi:10.1371/journal.pntd.0005680)

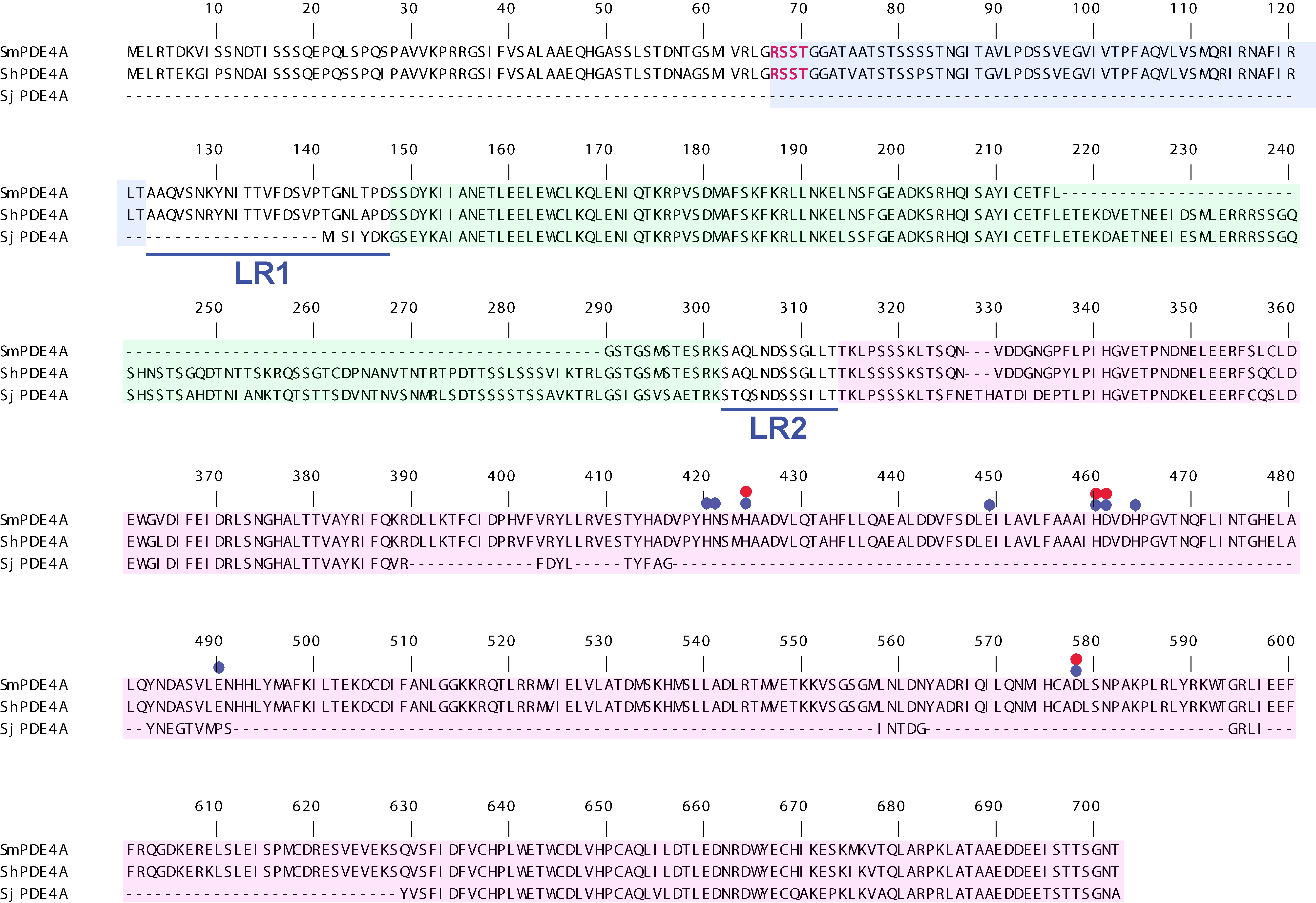

Supplement: S1 Fig — The alignment was generated using the PRABI (Pôle Rhône-Alpes de Bioinformatique) MULTALIN tool (https://npsa-prabi.ibcp.fr/). The image formatting is as presented in Fig 2. The Upstream Conserved Regions, UCR1 and UCR 2, and catalytic domains are indicated in blue, green and pink, respectively. The linker regions, LR1 and LR2, and the predicted PKA phosphorylation site in UCR1 are indicated. The conserved PDE signature motif HNX2HNXNE/D/QX10HDX2HX25E is indicated with blue circles and those residues that coordinate directly with the catalytic zinc in the substrate binding pocket are also indicated by the red circles. The gene identifiers for the S. mansoni, S. haematobium and S. japonicum sequences are Smp_134140, XM_012943524.1 and Sjp_0072560, respectively. (TIF) [file pntd.0005680.s001.tif]

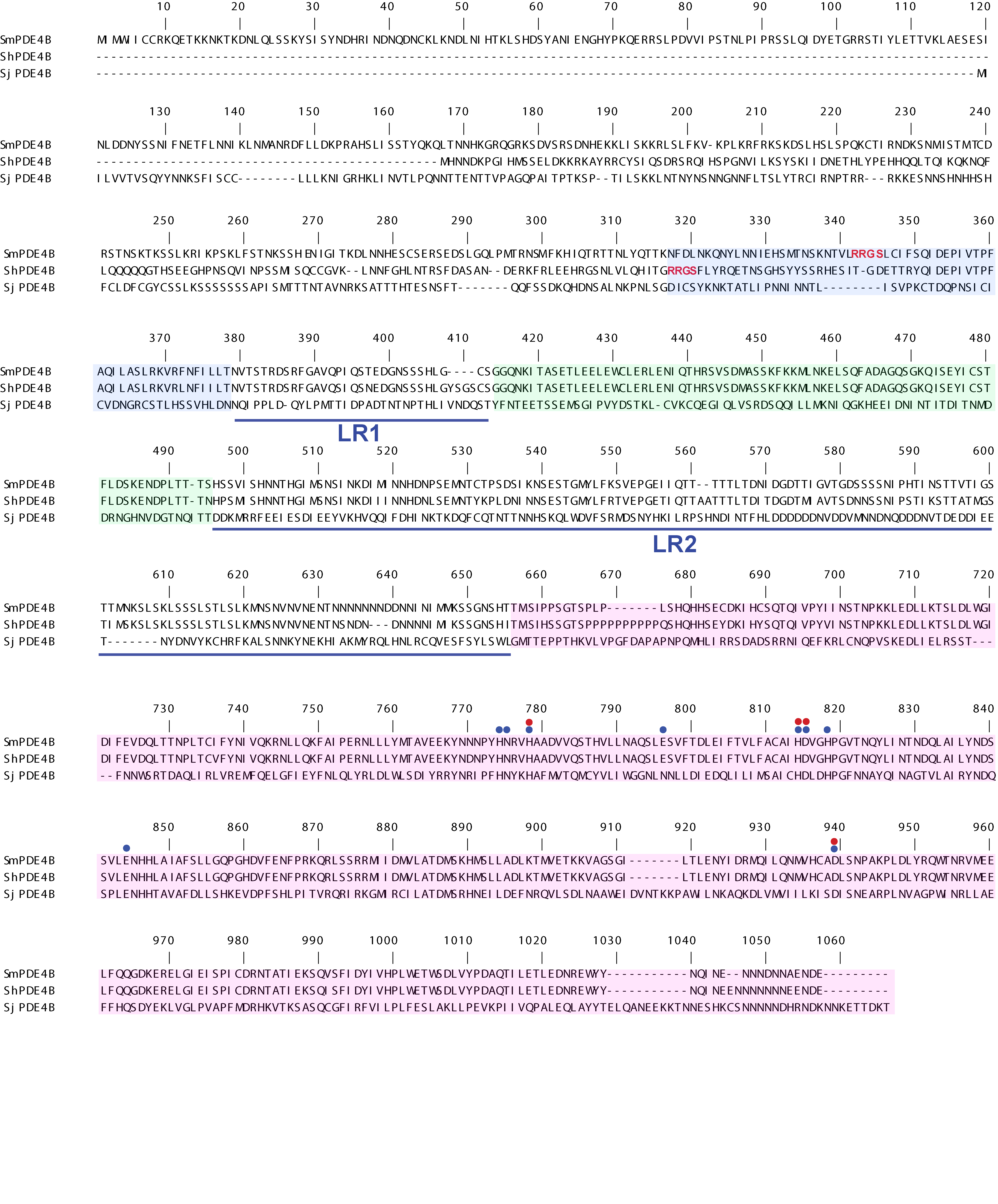

Supplement: S2 Fig — The alignment was generated using the PRABI (Pôle Rhône-Alpes de Bioinformatique) MULTALIN tool (https://npsa-prabi.ibcp.fr/). The image formatting is as presented in Fig 2. The Upstream Conserved Regions, UCR1 and UCR 2, and catalytic domains are indicated in blue, green and pink, respectively. The linker regions, LR1 and LR2, and the predicted PKA phosphorylation site in UCR1 are indicated. The conserved PDE signature motif HNX2HNXNE/D/QX10HDX2HX25E is indicated with blue circles and those residues that coordinate directly with the catalytic zinc in the substrate binding pocket are also indicated by the red circles. The gene identifiers for the S. mansoni, S. haematobium and S. japonicum sequences are Smp_141980, XM_012941682.1 and Sjp_0099480, respectively. (TIF) [file pntd.0005680.s002.tif]

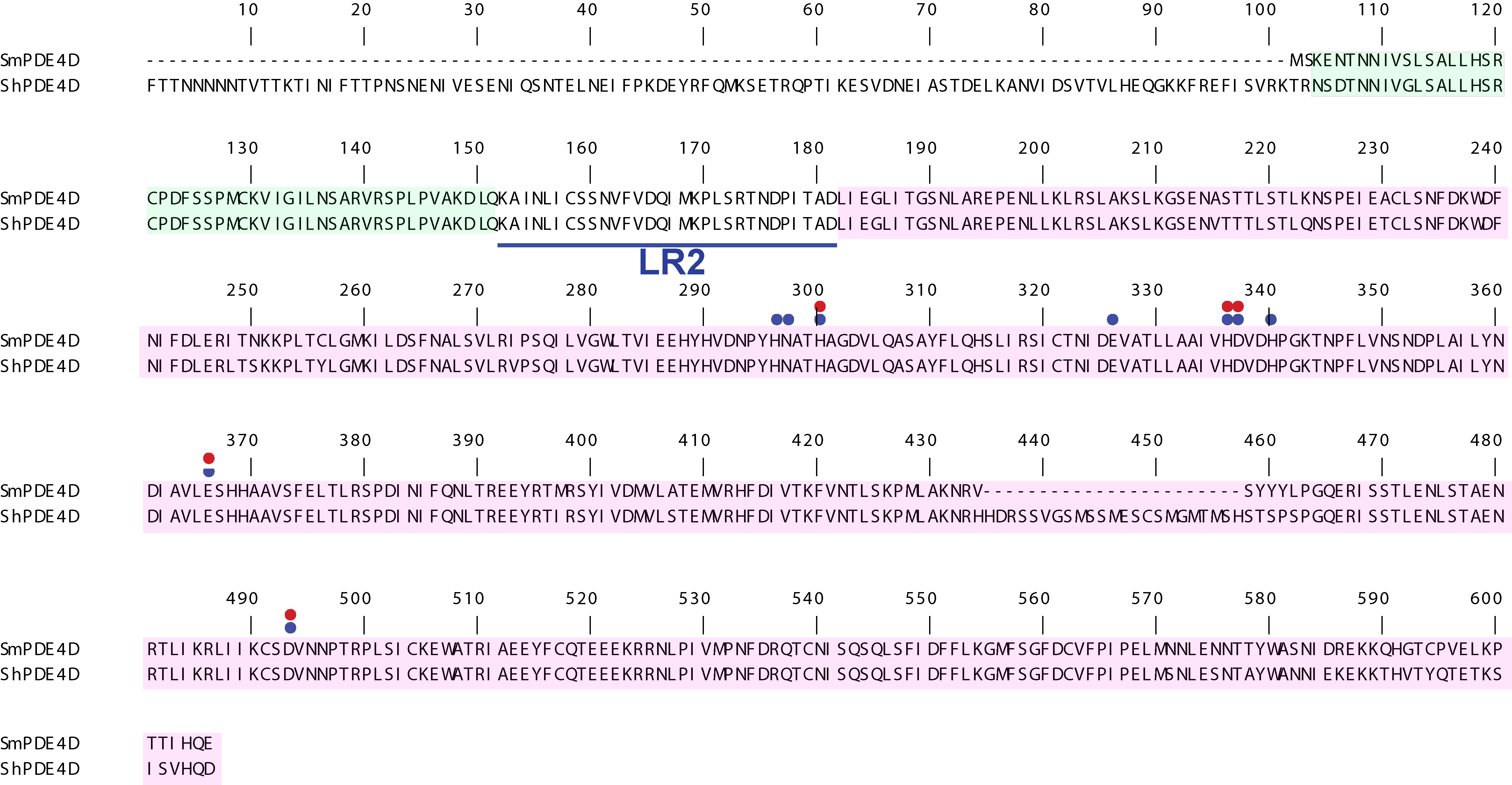

Supplement: S3 Fig — The alignment was generated using the PRABI (Pôle Rhône-Alpes de Bioinformatique) MULTALIN tool (https://npsa-prabi.ibcp.fr/). The image formatting is as presented in Fig 2. The Upstream Conserved Region, UCR 2, and the catalytic domain are indicated in green and pink, respectively: the linker region, LR2, is shown by the blue horizontal line. UCR1 and LR1 appear to be absent. The conserved PDE signature motif HNX2HNXNE/D/QX10HDX2HX25E is indicated with blue circles and those residues that coordinate directly with the catalytic zinc in the substrate binding pocket are also indicated by the red circles. The gene identifiers for the S. mansoni and S. haematobium sequences are Smp_044060 and XM_012937519.1, respectively. (TIF) [file pntd.0005680.s003.tif]

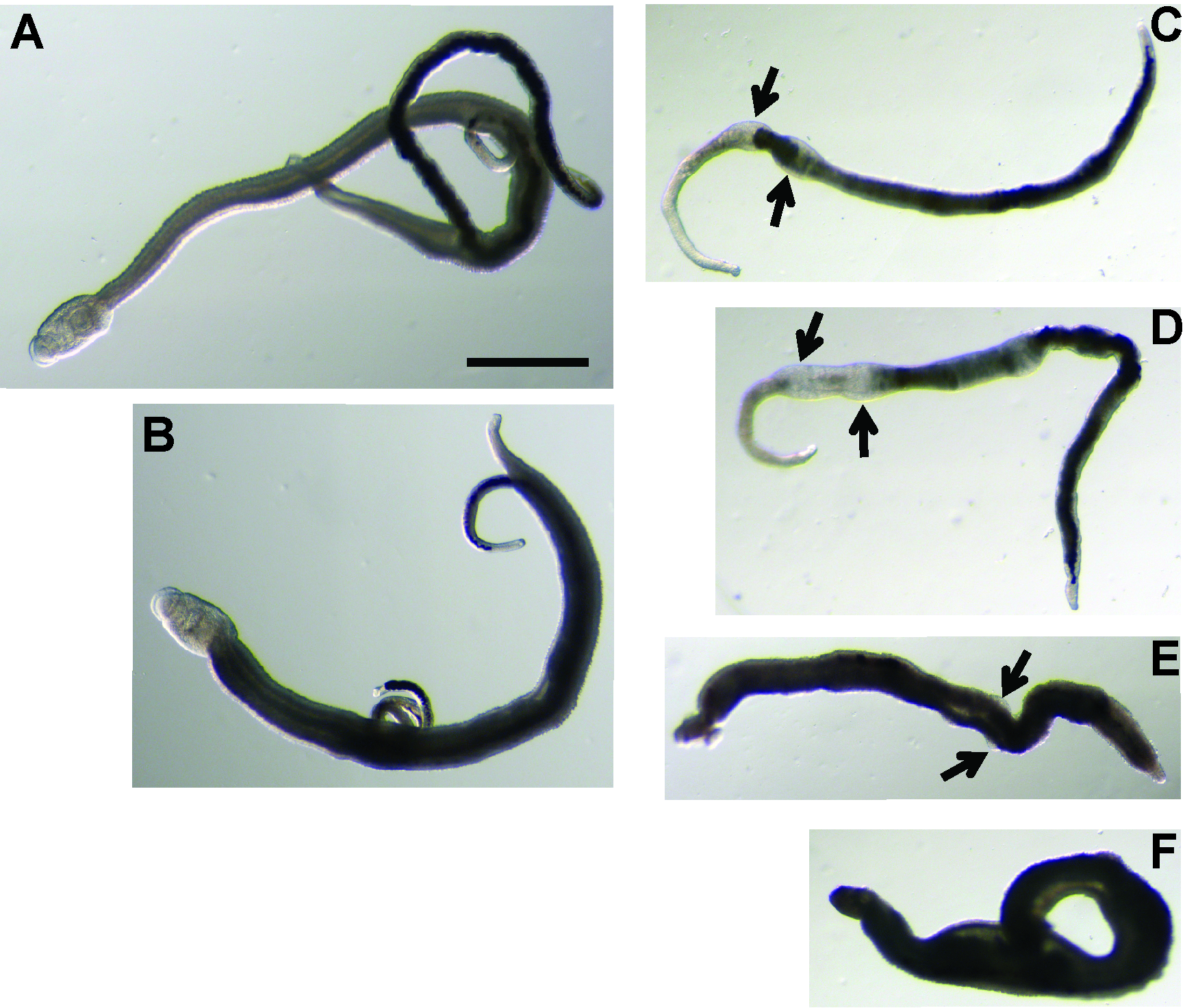

Supplement: S4 Fig — Parasites were incubated in the absence (A, B) or presence (C-F) of 10 μM compound 2 for 3 days as described in the text. The anterior of worms is leftmost in each panel. (A, B) Control worm pairs with males adhering to the well surface via the anterior oral and ventral suckers. The darker, thinner female is held within and is seen looping out from the male’s gynecophoral canal. (C, D) Female worms demonstrating bulging of the body wall (arrows) and a general derangement of body shape, particularly in D. Likewise, the male worms (E, F) have a deranged body shape: arrows in E point to areas of lifting (blebbing) of the tegument (surface). Bar = 150 μm. (TIF) [file pntd.0005680.s004.tif]
