# Supplementary material for: Phenotypic, chemical and functional characterization of cyclic nucleotide phosphodiesterase 4 (PDE4) as a potential anthelmintic drug target
Source: PLoS Negl Trop Dis. 2017 Jul 13;11(7):e0005680. doi: 10.1371/journal.pntd.0005680 (PMC5526615; doi:10.1371/journal.pntd.0005680)
Supplement: S1 Table — When information was not available regarding gene expression in a particular developmental stage, the corresponding cell was left blank. See text for details. (DOCX) [file pntd.0005680.s005.docx]

|  | ***S. mansoni*** | | | | | | ***S. haematobium*** | ***S. japonicum*** | |
| --- | --- | --- | --- | --- | --- | --- | --- | --- | --- |
|  | **Cercaria** | **Schistosomulum** | | **Adult** | **Adult Male** | **Adult Female** | **Adult** | **Schistosomulum** | **Adult Male** |
|  |  | **3 h** | **24 h** |  |  |  |  |  |  |
| **SmPDE4A** (Smp_134140, CCD81292.1) | X | X | X | X | X | X | XM_012943524.1 |  | (Sjp_0072560, AAX26934.2) |
| **SmPDE4B** Smp_141980, CCD80549.1) | X | X | X | X | X |  | XM_012941682.1 | (Sjp_0099480, AY811612.1) |  |
| **SmPDE4C**  (Smp_129270, CCD60676.1) | X | X | X | X |  |  |  |  |  |
| **SmPDE4D** (Smp_044060, CCD77807.1) | X | X | X | X |  |  | XM_012937519.1 |  |  |
