# Supplementary material for: Phenotypic, chemical and functional characterization of cyclic nucleotide phosphodiesterase 4 (PDE4) as a potential anthelmintic drug target
Source: PLoS Negl Trop Dis. 2017 Jul 13;11(7):e0005680. doi: 10.1371/journal.pntd.0005680 (PMC5526615; doi:10.1371/journal.pntd.0005680)
Supplement: S3 Table — A SmPDE4C ortholog was not found in S. haematobium, and SmPDE4C and D orthologs were absent in S. japonicum. (DOCX) [file pntd.0005680.s007.docx]

|  | | ***S. mansoni*** | | | | ***S. haematobium*** | | | ***S. japonicum*** | |
| --- | --- | --- | --- | --- | --- | --- | --- | --- | --- | --- |
|  |  | **PDE4A** | **PDE4B** | **PDE4C** | **PDE4D** | **PDE4A (XM_012943524.1)** | **PDE4B (XM_012941682.1)** | **PDE4D (XM_012937519.1)** | **PDE4A (Sjp_0072560)** | **PDE4B (Sjp_0099480)** |
| ***S. mansoni*** | **PDE4A** (Smp_134140) |  | 61 | 51 | 34 | 98 | 62 | 32 | 89 | 32 |
|  | **PDE4B** (Smp_141980) | 61 |  | 49 | 35 | 61 | 95 | 33 | 70 | 35 |
|  | **PDE4C**  (Smp_129270) | 51 | 49 |  | 32 | 51 | 51 | 29 | 47 | 34 |
|  | **PDE4D** (Smp_044060) | 34 | 35 | 32 |  | 34 | 35 | 87 | 34 | 31 |
| ***H. sapiens*** | **PDE4B2** (NP_001032416) | 65 | 63 | 51 | 34 | 64 | 63 | 30 | 61 | 33 |
| ***C. elegans*** | NP_495601 | 58 | 61 | 51 | 32 | 57 | 61 | 30 | 53 | 33 |
